# Genetic variability in response to amyloid beta deposition influences Alzheimer’s disease risk

**DOI:** 10.1093/braincomms/fcz022

**Published:** 2019-10-10

**Authors:** Dervis A Salih, Sevinc Bayram, Sebastian Guelfi, Regina H Reynolds, Maryam Shoai, Mina Ryten, Jonathan W Brenton, David Zhang, Mar Matarin, Juan A Botia, Runil Shah, Keeley J Brookes, Tamar Guetta-Baranes, Kevin Morgan, Eftychia Bellou, Damian M Cummings, Valentina Escott-Price, John Hardy

**Affiliations:** 1 Department of Neuroscience, Physiology and Pharmacology, UCL, Gower Street, London WC1E 6BT, UK; 2 UK Dementia Research Institute at UCL, Gower Street, London WC1E 6BT, UK; 3 Hitachi Rail Europe Ltd, Holborn, London, UK; 4 Department of Neurodegenerative Diseases, Institute of Neurology, UCL, 1 Wakefield Street, London WC1N 1PJ, UK; 5 Department of Information and Communications Engineering, Universidad de Murcia, Spain; 6 Human Genetics, School of Life Sciences, Life Sciences Building, University Park, University of Nottingham, Nottingham NG7 2RD, UK; 7 Dementia Research Institute, MRC Centre for Neuropsychiatric Genetics and Genomics, Cardiff University, UK

**Keywords:** Alzheimer’s, microglia, amyloid, genome-wide association studies, expression quantitative trait loci

## Abstract

Genome-wide association studies of late-onset Alzheimer’s disease risk have previously identified genes primarily expressed in microglia that form a transcriptional network. Using transgenic mouse models of amyloid deposition, we previously showed that many of the mouse orthologues of these risk genes are co-expressed and associated with amyloid pathology. In this new study, we generate an improved RNA-seq-derived network that is expressed in amyloid-responsive mouse microglia and we statistically compare this with gene-level variation in previous human Alzheimer’s disease genome-wide association studies to predict at least four new risk genes for the disease (*OAS1*, *LAPTM5*, *ITGAM/*CD11b and *LILRB4*). Of the mouse orthologues of these genes *Oas1a* is likely to respond directly to amyloid at the transcriptional level, similarly to established risk gene *Trem2*, because the increase in *Oas1a* and *Trem2* transcripts in response to amyloid deposition in transgenic mice is significantly higher than both the increase of the average microglial transcript and the increase in microglial number. In contrast, the mouse orthologues of *LAPTM5*, *ITGAM*/CD11b and *LILRB4* (*Laptm5*, *Itgam*/CD11b and *Lilra5*) show increased transcripts in the presence of amyloid plaques similar in magnitude to the increase of the average microglial transcript and the increase in microglia number, except that *Laptm5* and *Lilra5* transcripts increase significantly quicker than the average microglial transcript as the plaque load becomes dense. This work suggests that genetic variability in the microglial response to amyloid deposition is a major determinant for Alzheimer’s disease risk, and identification of these genes may help to predict the risk of developing Alzheimer’s disease. These findings also provide further insights into the mechanisms underlying Alzheimer’s disease for potential drug discovery.

## Teaser

How genetic loci for determining risk of Alzheimer’s disease confer their effects is unclear. Salih *et al.* demonstrate risk genes are connected in a microglial transcriptional network. This network overlaps with human gene variation, indicating new risk genes. This will allow better prediction of disease and implicates new drug targets.

## Graphical Abstract

**Graphical Abstract:**
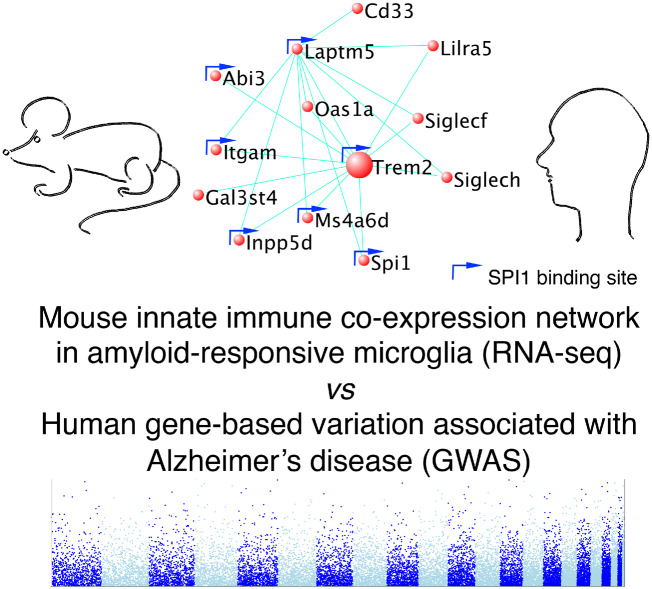

## Introduction

All the known mutations in genes causing early-onset Alzheimer’s disease alter amyloid precursor protein (APP) processing such that amyloid deposition becomes more likely (Hardy and Selkoe, 2002). In contrast, despite some rare variants in APP processing enzymes (Kim *et al.*, 2009; Marioni *et al.*, 2018; Jansen *et al.*, 2019; Kunkle *et al.*, 2019), the majority of the risk in late-onset disease has been shown to be due to sequence variability in genes expressed in the innate immune system (largely microglial genes; Jones *et al.*, 2010). We and others identified the microglial gene *TREM2* as a potent risk gene for late-onset disease (Guerreiro *et al.*, 2013; Jonsson *et al.*, 2013), and identified that its expression was strongly increased by amyloid deposition in *APP* transgenic mice (Guerreiro *et al.*, 2013; Matarin *et al.*, 2015; Cheng-Hathaway *et al.*, 2018; Song *et al.*, 2018). We previously reported a microarray analysis of genome-wide expression of a range of transgenic mice expressing mutant human *APP* and/or *PSEN1* (Matarin *et al.*, 2015). The different lines of mice analysed in this study developed amyloid plaques at different rates and so, by analysis of plaque deposition and gene expression in the same animals, plaque deposition could be correlated with gene expression across the life of a mouse, independent of age. We noted that *Trem2* was one of the genes whose expression was up-regulated the most in relation to amyloid deposition. *Trem2* expression also showed a strong correlation with the expression of a network of genes in the innate immune system suggesting *Trem2* is a ‘hub’ gene, and may regulate the expression of the entire network. This immune module of genes showed a remarkable positive correlation to amyloid deposition (Matarin *et al.*, 2015), and contained orthologues of other already established Alzheimer’s disease risk genes such as *Abca7* and *Ms4a6d* (Lambert *et al.*, 2013). Notably, two genes, *ABI3* and *PLCG2*, that were identified subsequently as being associated with Alzheimer’s disease risk loci (Sims *et al.*, 2017), were also present in this network. Hence, mouse microglia clearly respond to plaques in a manner where the genes co-expressed within these microglia relate closely to the genes that are relevant in human disease. These observations also suggest that this innate immune network that is expressed by these amyloid-responsive microglia may be used to predict future risk genes for Alzheimer’s disease.

An important outstanding question is whether progression of late-onset Alzheimer’s disease to the point of neurodegeneration and diagnosis is largely due to an inadequate innate immune response to rising amyloid beta (Aβ) deposition, resulting in accelerated amyloid-induced damage (Edwards, 2019). This hypothesis is difficult to study in human post-mortem tissue because during pathogenesis the proportion of cell types in the brain changes and the remaining cells show extensive compensatory changes in gene expression. With this in mind, for this new work we developed the approach outlined below to use the gene expression network that is present within amyloid-responsive microglia in mouse models during pathology progression, and tested for significant overlap with human gene variation associated with Alzheimer’s disease. We then surveyed the gene expression network in mouse amyloid-responsive microglia to investigate if we could identify further Alzheimer’s disease risk loci. Initially, we took advantage of the increased resolution provided by performing RNA-seq to improve the gene expression analysis we had previously undertaken with microarray technology in the same mice. The new higher-resolution transcriptional network containing the co-expressed mRNA that most strongly correlated to amyloid deposition again featured primarily microglial genes. This confirmed the previous analysis in the same mice (Matarin *et al.*, 2015), but the mouse RNA-seq analysis revealed many additional genes not detectable with microarray, and included yet more genes previously identified as human risk genes for Alzheimer’s disease from genome-wide association studies (GWAS). We then investigated whether the genes included in the novel co-expression network present in amyloid-responsive mouse microglia are also significantly associated with Alzheimer’s disease in human GWAS data. We used the data from the International Genomics of Alzheimer’s Projects (IGAP; Lambert *et al.*, 2013; Kunkle *et al.*, 2019) to identify the genes which are present in the mouse network and also significantly associated with Alzheimer’s disease risk. The significance of each human gene was assessed using a gene-based approach, applied to the summary statistics of the IGAP datasets (Brown, 1975; Moskvina *et al.*, 2011; Escott-Price *et al.*, 2014; de Leeuw *et al.*, 2015). The gene-based analyses employed here account for the strength of the association of multiple adjacent single nucleotide polymorphisms (SNPs), restricted to the exon boundaries of genes. This approach has important implications for predicting disease risk in people at the gene level (rather than SNP-level), with the potential of providing mechanistic insights into the cellular and molecular processes underlying disease progression.

## Materials and methods

### Mouse models of Alzheimer’s disease

The RNA samples used for this study were from the same mice we used previously, described in detail in Matarin *et al.* (2015), therefore no further mice were bred for this study. The mouse lines used are stated in the Supplementary material. The mice procedures used for Matarin *et al.* (2015), were performed in agreement with the UK Animals (Scientific Procedures) Act, 1986, with local ethical agreement.

### Human genome-wide association studies data

The original IGAP (Lambert *et al.*, 2013) summary statistics calculated for each SNP with 17 008 Alzheimer’s disease cases and 37 154 controls (Stage 1) were used to derive the gene-based *P*-values, described further below and in Escott-Price *et al.* (2014). The updated IGAP (Kunkle *et al.*, 2019) summary statistics, derived from 21 982 clinically confirmed Alzheimer’s disease cases and 41 944 controls (Stage 1) were used to repeat the procedure and generate gene-based *P*-values to determine if the associations identified from the original IGAP data remained.

### Mouse transcriptome work

For this study, RNA-seq library preparation and sequencing was performed by Eurofins Genomics (Ebersberg, Germany), details given in Supplementary material together with processing of FASTQ files. Supplementary Fig. 1 shows how the new RNA-seq data and new comparison to IGAP GWAS data for Alzheimer’s disease, relates to total RNA samples collected previously in Matarin *et al.* (2015).

Weighted gene co-expression network analyses were performed as described in Matarin *et al.* (2015), using the recommended parameters from the original analysis developers (Zhang and Horvath, 2005; Horvath *et al.*, 2006; Oldham *et al.*, 2006; Langfelder and Horvath, 2008). Further details are in Supplementary material.

### Gene-based human genome-wide association studies data analysis

The significance of the association to Alzheimer’s disease of human genes was assessed using a gene-based approach as introduced in Brown (1975), Escott-Price *et al.* (2014), and implemented in de Leeuw *et al.* (2015; MAGMA software ctg.cncr.nl/software/magma). Briefly, the updated IGAP (Kunkle *et al.*, 2019) summary statistics calculated for each SNP in a sample of 21 982 Alzheimer’s disease cases and 41 944 controls were used to derive gene-based *P*-values. SNPs were assigned to genes if they were located within the genomic sequence corresponding to the start of the first and the end of the last exon of each transcript. Previous analyses including the 10 kb sequence flanking the first and last exons of genes, which may contain potential regulatory SNPs, did not cause substantial differences to the gene-based *P*-values (Escott-Price *et al.*, 2014). Gene locations were as Build 37, Assembly Hg19 of the National Center for Biotechnology Information (NCBI) database as provided as part of the MAGMA software package. Phase 3 of 1000 Genomes data were used as a reference panel for calculation of linkage disequilibrium between markers (Genomes Project Consortium *et al.*, 2015). The gene-wide analysis was performed based on the summary *P*-values while controlling for linkage disequilibrium and different numbers of SNPs per gene using a statistical approach by Brown (1975), and adopted for set-based analysis of genetic data by Moskvina *et al.* (2011) and de Leeuw *et al.* (2015). Prior to the gene-based analyses all individual SNP *P*-values were corrected for the genomic inflation factor (λ; to normalise for unaccounted variation, due to factors such as population stratification; Devlin and Roeder, 1999).

### Statistical analysis comparing human genes with co-expression network of amyloid-responsive mouse microglia

The lists of mouse genes in the co-expression networks were converted to lists of human gene names using convertMouseGeneList() function, library biomaRt in R downloaded from https://bioconductor.org/biocLite.R. We tested whether the *number* of Alzheimer’s disease associated genes (at significance thresholds alpha = 0.05, 0.01 and 0.001) in the mouse co-expression network was greater than that expected by chance given the number of human orthologues present in the mouse network. For that, we counted the observed number of independent significant human genes in the mouse network and compared this with the expected (by chance) number of genes calculated as N*alpha, whilst accounting for the variance (var=N*alpha*(1‐alpha)), where *N* was the total number of independent human genes in the mouse network. To account for linkage disequilibrium, the genes within 0.5 Mb of each other were conservatively counted as one. The *P*-value of the excess of significant genes in the mouse network, between observed and expected, was calculated using a *Z*-test comparing the number of observed significant genes with the expected number. The observed number of significant genes was significantly higher than the expected at all gene *P*-value thresholds (0.05, 0.01, 0.001) for the amyloid-associated network. We report the genes at the gene-based *P*-values at threshold alpha = 0.01.

### Data availability statement

RNA-seq expression data have been deposited in NCBI’s Gene Expression Omnibus (GEO; Series accession number GSE137313; https://www.ncbi.nlm.nih.gov/geo/query/acc.cgi?acc=GSE137313), and are available at: www.mouseac.org.

## Results

### High-resolution co-expression network using RNA-seq in amyloid-responsive microglia

**Figure 1:**
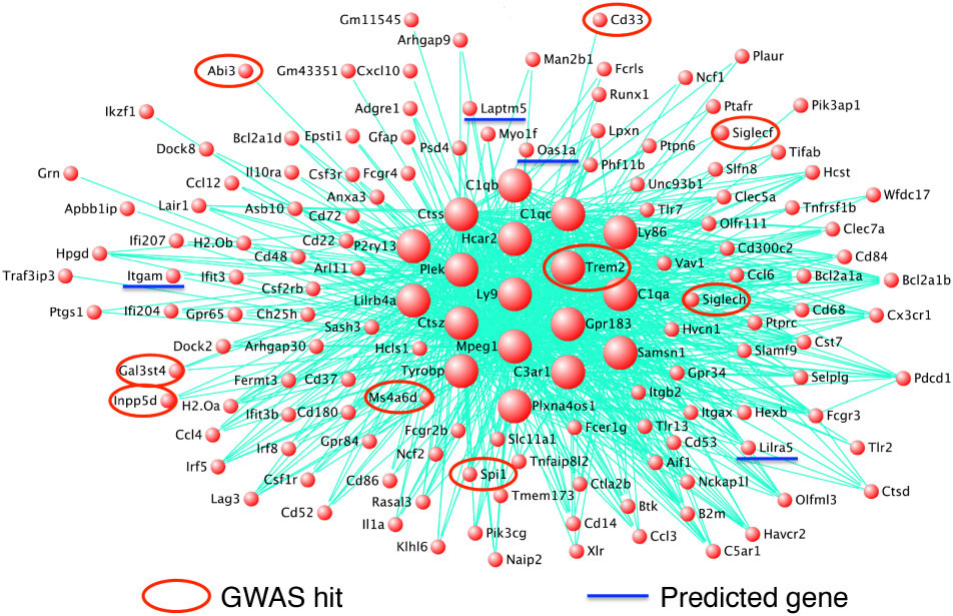
**An innate immune network of genes expressed by amyloid-responsive microglia, featuring several orthologues of established GWAS genes associated with Alzheimer’s disease, predicts the importance of four new risk genes that may influence the risk of developing Alzheimer’s disease.** Network plot using VisANT reveals key drivers of an innate immune module from RNA-seq derived gene expression from the hippocampus of wild-type and amyloid mice. Red circles show orthologues of established GWAS genes associated with Alzheimer’s disease including *Trem2*, *Abi3*, *Cd33* and *Spi1*/PU.1. Blue underline shows gene orthologues predicted to confer altered risk of Alzheimer’s disease by overlapping a gene co-expression network present in mouse microglia that show a strong response to amyloid in transgenic mice with individual human genes significantly associated with Alzheimer’s disease by analysing combinations of adjacent SNPs (see Materials and methods section; Escott-Price *et al.*, 2014). Genes shown in this network are transcribed and co-expressed in amyloid-responsive microglia. Larger red spheres represent ‘hub’ genes, those showing the greatest number of connections to other genes in the network, and include *Trem2*, *Tryobp*, *Lilrb4a*, *P2ry13*, *Ctss*, *Ctsz*, *Mpeg1* and *Plek*, which are likely to play important roles in driving microglial function.

Although mouse models for dementia have clear limitations in that they do not show tau tangles or neuronal loss solely in response to rising Aβ, they allow us to study the time-course response of a healthy innate immune system reacting to Aβ, leading to the possibility that the innate immune cells of the mouse may ultimately be preventing Aβ killing neurons. We previously constructed a transcriptional network using expression arrays that was present in microglia that respond to plaques (Matarin *et al.*, 2015). As microarrays are limited by their probe content and their dynamic range, for this new study we have now sequenced the transcriptome of the same mice, expressing one or two copies of the *APP* (Swedish) and/or *PSEN1* (M146V) transgenes alongside wild-type controls, using RNA-seq to construct a new higher resolution expression network. Similar to our findings with the initial microarray analysis, the RNA-seq analysis revealed a microglial module of genes that showed a strong correlation with Aβ deposition (correlation = 0.94; *P* < 3e^−41^), and contained the mouse orthologues of the identified GWAS loci *TREM2*, *ABI3*, *CD33*, *INPP5D*, *MS4A6D*, *SPI1/*PU.1, *PLCG2*, *GAL3ST4*, *RIN3, HLA* and *APOE* (Supplementary Table 1), verifying the relevance of this gene network to the human condition. Our hypothesis is that this network contains most of the genes that the microglia need to respond to amyloid plaques, including genes necessary for increases in cell number and activation (thus many cellular responses including proliferation, survival, metabolism, activation into a variety of states and phagocytosis). The genes showing the tightest expression correlation within the module associated with microglia reacting to plaques form the network shown in Fig. 1 and Supplementary Table 2 (top 147 genes from a total of 1584 genes expressed as part of the innate immune module based on the topological overlap measure, connectivity values). This network is broadly similar to the network derived from the analysis of the same RNA by microarray methods (Matarin *et al.*, 2015) and shows common features with microglial networks published by other groups using other amyloid mouse models (Wang *et al.*, 2015; Castillo *et al.*, 2017; Keren-Shaul *et al.*, 2017; Lee *et al.*, 2018; Nam *et al.*, 2018), suggesting this is a conserved core network of genes that can be reliably identified using different methodologies. *Trem2* forms a hub gene in our network, using either technique, indicating that *Trem2* expression is highly correlated to many other genes in the network, and may drive the response of this network. In line with this idea, *Trem2* has been shown to regulate at least part of this immune module (Wang *et al.*, 2015; Keren-Shaul *et al.*, 2017; Lee *et al.*, 2018). The network we identified is also broadly similar to a human network of innate immune genes containing *TYROBP*, *TREM2*, *MS4A* family genes, *C1Q* members and *CD33*, identified from human post-mortem tissue bearing in mind the caveats discussed above for human tissue (Forabosco *et al.*, 2013; Zhang *et al.*, 2013). Again this suggests that this gene network expressed by Aβ-responsive mouse microglia behaves similarly in humans.

### Enrichment of human Alzheimer’s disease genes in the mouse gene network expressed by amyloid-responsive microglia

Traditionally, GWAS projects have focused on single SNPs because single locus tests are the easiest to test and interpret, but these have limitations. For example, if disease risk is conferred by several (semi) independent SNPs within a locus with moderate effect sizes, this locus (gene) will be overlooked by the genome-wide analyses, as the statistical significance of each individual SNP will not pass the Bonferroni correction. Therefore, if only single SNPs are considered, useful disease associations may be lost, despite apparently high sample sizes (Escott-Price *et al.*, 2014). To identify genes associated with Alzheimer’s disease at the gene-based level we initially used the summary statistics from the original IGAP (Lambert *et al.*, 2013), and then re-ran the gene-based analyses using the larger updated IGAP data (Kunkle *et al.*, 2019). We considered multiple SNPs within individual human genes to generate gene-level *P*-values in order to assess whether multiple SNPs together constitute a significant risk factor, using a gene-based approach applied to the Alzheimer’s disease GWAS summary statistics (Brown, 1975; Escott-Price *et al.*, 2014; de Leeuw *et al.*, 2015). Within our mouse innate immune network, we first confirmed the significance of several members of the network that were orthologues of established Alzheimer’s disease loci variants using the gene-level *P*-values, including genes such as *Trem2* and *Abi3* (Table 1). We then asked whether the other members of the mouse network expressed by amyloid-responsive microglia might predict additional risk for Alzheimer’s disease. To this end, we identified orthologues of human genes in the mouse network and tested whether this set of genes is enriched for the genes which contain variants significantly associated with Alzheimer’s disease. As this set of genes was defined by our biological experiment in contrast to genome-wide analyses, which by their nature are exploratory rather than hypothesis driven, we considered a nominal statistical significance threshold of *P* = 0.05 for human Alzheimer’s disease gene-based associations. We also explored more stringent significance thresholds (*P* = 0.01 and *P* = 0.001), for selection of the genes for the gene enrichment analysis. To ensure that our enrichment analysis results were not inflated by the correlated genes due to linkage disequilibrium (i.e. in close proximity to one another), the genes within 0.5 Mb of each other were counted as one. We found a significant enrichment of orthologues of human genes associated with Alzheimer’s disease at the *P* = 0.01 significance threshold within this mouse network expressed by amyloid-responsive microglia over and above that expected by chance (*P* = 8.86 × 10^−6^). The enrichment remained significant even after the established GWAS loci were excluded [*P* = 1.66 × 10^−4^ for highly connected network of 147 genes (Fig. 1) and similarly *P* = 3.68 × 10^−4^ for the entire module of 1584 genes (Supplementary Table 1)]. GWAS loci boundaries were defined as 0.5 Mb from either side of the most significant SNPs of previously identified GWAS genes with exclusion of APOE and HLA which we defined as chromosome 19: 44 500 000−46 500 000 and chromosome 6: 32 200 000 − 32 800 000, respectively.

**Table 1:** The genes predicted to contain variants associated with Alzheimer’s disease together with established loci from GWAS

Mouse symbol (MGI)	Human symbol (HGNC)	NCBI ID	Human chromo- some	Start location	End location	Number of SNPs	Gene *P*-value (adj for GC)	Best SNP	Best SNP Location	Best SNP p-value	Effect size	Risk Allele	Frequency
Predicted genes													
* Laptm5*	*LAPTM5*	7805	1	31205316	31230667	71	6.62E−05	rs7549164	31224193	4.15E−04	0.0655	T	0.1935
* Oas1a*	*OAS1*	4938	12	113344582	113371027	126	1.58E−03	rs4766676	113365581	6.16E−04	0.0518	T	0.6209
* Itgam*	*ITGAM*	3684	16	31271288	31344213	168	4.92E−03	rs79113991	31273662	4.48E−03	0.0656	A	0.1308
* Lilra5*	*LILRB4*	11006	19	55155340	55181810	148	8.96E−03	rs731170	55176262	1.72E−03	0.0513	A	0.3023
Established GWAS genes													
* Inpp5d*	*INPP5D*	3635	2	233924677	234116549	720	9.81E−06	rs10933431	233981912	2.55E−07	0.1001	C	0.7774
* Trem2*	*TREM2*	54209	6	41126244	41130924	5	1.47E−08	rs7748513	41127972	1.81E−03	−0.1175	A	0.9617
* Gal3st4*	*GAL3ST4*	79690	7	99756867	99766373	21	4.68E−03	rs34130487	99759205	3.47E−03	−0.0474	T	0.2811
* Spi1*	*SPI1*	6688	11	47376411	47400127	87	8.96E−12	rs3740688	47380340	9.70E−11	0.0935	T	0.5524
* Ms4a6d*	*MS4A6A*	64231	11	59939487	59952139	33	2.10E−12	rs7935829	59942815	6.78E−15	0.1134	A	0.5979
* Abi3*	*ABI3*	51225	17	47287589	47300587	47	4.93E−02	rs9896800	47293329	8.62E−03	0.0417	T	0.6772
* Cd33*	*CD33*	945	19	51728320	51747115	34	1.09E−06	rs12459419	51728477	4.51E−07	−0.0800	T	0.3076

Genes predicted to confer altered risk of Alzheimer’s disease by overlapping gene expression data transcribed by microglia that show a strong response to plaques in amyloid mice (Fig. 1) with individual human genes significantly associated with Alzheimer’s disease by analysing combinations of adjacent SNPs (see Materials and methods section; Escott-Price *et al.*, 2014). The SNP data were from the updated IGAP study, using Build 37, Assembly Hg19 (Kunkle *et al.*, 2019). The SNP with the most significant p-value within each gene is denoted as ‘Best SNP,’ and is stated for completion from the updated IGAP stage 1 dataset, but was not used for any statistical calculations in this manuscript. The effect size (coefficient of the logistic regression) is provided for the best reported SNP from IGAP data; a positive number indicates that the allele increases risk of Alzheimer’s disease, and so a negative number indicates the allele is protective. The allele frequency from the IGAP study is also provided. The established genes altering risk for Alzheimer’s disease from GWAS are given for comparison.

In contrast to the mouse gene network expressed by amyloid-responsive microglia, the innate immune network expressed by microglia responding to tau pathology in mice transgenic for tau (P301L), was not significantly enriched for human genes associated with Alzheimer’s disease using the same methods (*P* = 0.78), although *Apoe* is part of this module and this module also contained genes largely expressed by microglia (Supplementary Fig. 2, top 137 genes from a total of 2299 genes in the module based on the topological overlap measure). When the entire module of innate immune genes expressed by tauopathy-responsive microglia (2299 genes) was considered there was a modest significant enrichment, *P* = 1.74 × 10^−2^, suggesting that a proportion of genes associated with Alzheimer’s disease through multiple SNPs are microglial genes that have mouse orthologues, but are expressed by microglia that are less responsive to tau pathology compared to Aβ deposition.

The analysis of the genetic network expressed by amyloid-responsive microglia identified five genes within the central portion of mouse microglial network whose human orthologues were associated with Alzheimer’s disease from the original IGAP data [described in Salih *et al.* (2018), using the IGAP data from Lambert *et al.* (2013)]. When we repeated the analysis using the updated IGAP data (Kunkle *et al.*, 2019) containing 29.2% more cases and 12.9% more controls, and 62.7% more SNPs as compared to Lambert *et al.* (2013), four of the five identified genes from the centre of the co-expression network in mice were still strongly associated with the orthologues containing variants in human Alzheimer’s disease. These four genes, *OAS1*, *LAPTM5*, *ITGAM/*CD11b and *LILRB4*, have not been previously reported as having variants significantly associated with Alzheimer’s disease using traditional GWAS approaches (Table 1, Supplementary Figs 3 and 4). In addition, amongst the entire genetic network expressed by amyloid-responsive microglia (Supplementary Table 1; 1584 genes), a further 12 mouse genes have orthologues associated with human Alzheimer’s disease (*P* < 0.01) from the updated IGAP study (Supplementary Table 3). We emphasise that the goal of this comparison between the genetic network in mouse amyloid-responsive microglia versus human genes associated with Alzheimer’s disease combining multiple SNPs in a given gene was not to identify new single SNPs with genome-wide significant *P*-values ≤5 × 10^−8^. Instead, the alternative approaches we describe here were used to survey for more complex relationships between DNA variation and coding genes associated with Alzheimer’s disease by: (1) selecting a network of biologically relevant genes to Alzheimer’s disease genes (which reduces dramatically the number of genes being surveyed, to 1584 genes in our amyloid-associated network); (2) considering all SNPs together bounded by the coding region of a given gene (the gene-based analysis); and (3) looking at the network as a whole rather than individual genes (the enrichment analysis). Hence the individual gene significance is modest as compared to the genome-wide levels, but the genes are statistically significant and, together with previously identified Alzheimer’s disease genes, form the core of a transcriptional gene network (Fig. 1 and Table 1).

If we consider a sub-network of genes expressed by amyloid-responsive microglia that contains these four novel putative risk genes with the established GWAS loci *TREM2*, *ABI3*, *CD33*, *INPP5D, SPI1*/PU.1, *MS4A6D* and *GAL3ST4* present in Fig. 1, this sub-network is not highly connected in an innate immune gene network associated with tauopathy (Supplementary Fig. 2), suggesting this sub-network is expressed by microglia that are more responsive to amyloid deposition than other pathological features. Furthermore, in common with the existing seven known GWAS-associated genes in Fig. 1, the four novel risk genes we identify that are expressed by microglia that respond to Aβ deposition show transcript levels rising from 4 months of age in the homozygous *APP/PSEN1* mice and after 4 months of age in the hemizygous *APP/PSEN1* mice (Supplementary Fig. 5), in line with the increase in microglial numbers as amyloid plaques begin to deposit (Medawar *et al.*, 2019). To investigate whether the transcriptional changes we observed here are due to the increased microglial numbers in response to amyloid plaques we observed previously (Medawar *et al.*, 2019), and to determine which genes are directly up-regulated or down-regulated by amyloid at the mRNA level beyond the changes in microglial number, we calculated fold change of each gene in the homozygous and hemizygous *APP/PSEN1* mice relative to its expression in age-matched wild-type mice (Supplementary Fig. 6). The expression levels of our putative risk genes relative to expression in age-matched wild-type mice shows a range (*Oas1a*, 10.0-fold increase in homozygous *APP/PSEN1* mice relative to wild-type at 18 months of age; *Laptm5*, 4.1-fold increase; *Lilra5*, 3.8-fold increase; *Itgam*/CD11b, 2.3-fold increase; compared to *Trem2*, 9.2-fold increase, and *Aif1*, 3.3-fold increase; Supplementary Fig. 6). Genes showing higher relative transcript levels such as *Oas1a* and *Trem2* compared to the average transcript level relative to wild-type mice for the entire innate immune network throughout disease progression, thus are likely to be directly up-regulated in response to amyloid by the reacting microglia, considering the number of microglia (3.7-fold increase in microglia at 18 months of age in homozygous *APP/PSEN1* mice compared to wild-type; Medawar *et al.*, 2019). In contrast, *Laptm5* and *Lilra5* relative expression are only significantly increased relative to average transcript level of the entire network when the plaque load starts to become heavy (8 months of age), but returns to the average relative transcript level of the network as disease progresses, suggesting a role in the initial response to Aβ (Supplementary Fig. 6). *Itgam*/CD11b shows a similar change in relative expression to the average relative transcript level of the entire immune network, and to the increase in microglia numbers, comparable to relative *Spi1*/PU.1 expression, suggesting that *Itgam*/CD11b *and Spi1*/PU.1 transcription is unlikely to be directly regulated by Aβ, but may play a role in regulating the change in microglia number in response to amyloid plaques because of the strong correlation between pathology and *Itgam*/CD11b expression. The expression patterns for *Oas1a*, *Lilra5* and *Itgam*/CD11b are similar in both the homozygous and hemizygous *APP/PSEN1* mice (Supplementary Fig. 6), whereas *Laptm5* shows an expression pattern in the hemizygous *APP/PSEN1* mice that is more similar to *Itgam*/CD11b. The similarity of the expression profiles of *Laptm5*, *Itgam*/CD11b and *Spi1*/PU.1 in the hemizygous *APP/PSEN1* mice suggests that these three genes may play a role in regulating microglial number in response to amyloid deposition.

### Transcriptional network expressed by amyloid-responsive microglia containing risk genes is conserved in humans

Aspects of the transcriptional network associated with amyloid that we identified in our analysis, containing the four predicted risk genes with the existing seven GWAS loci, are broadly similar to microglial networks we and others have previously identified in human brain analyses. Zhang *et al.* identified an Alzheimer’s disease-relevant network centred on *TYROBP* and *TREM2*, which contained *ITGAM*/CD11b and *LAPTM5* (Zhang *et al.*, 2013), and we described a human microglial network containing *LAPTM5*, *ITGAM*/CD11b and *LILRB4* (Forabosco *et al.*, 2013). We then determined whether these novel Alzheimer’s disease risk genes, derived from a mouse transcriptional network expressed by amyloid-responsive microglia were present in independent datasets of human brain co-expression networks. Cross referencing our network with the data from the ROS/MAP project (Bennett *et al.*, 2012a, *b*; De Jager *et al.*, 2018), revealed that *LAPTM5*, *ITGAM*/CD11b and *LILRB4* clustered together with many of the GWAS risk genes for Alzheimer’s disease (Supplementary Fig. 7; Fisher’s Exact test Bonferroni corrected *P* = 1.34 × 10^−13^ showing a significant overlap between the genes in the mouse amyloid-associated module and human genes in the ROS/MAP module associated with Alzheimer’s disease). Interestingly, *SPI1*/PU.1, the myeloid cell transcription factor and a newly discovered GWAS risk gene (Huang *et al.*, 2017) was also in the same ROS/MAP module as *LAPTM5*, *ITGAM*/CD11b and *LILRB4*. We confirmed these module memberships in the BRAINEAC data for non-Alzheimer’s disease control human brains generated in our own lab (Ramasamy *et al.*, 2014). Interestingly, we found that SPI1/PU.1 binds to the regulatory regions of *Laptm5* and *Itgam*/CD11b, as well as established Alzheimer’s disease risk gene orthologues *Trem2*, *Abi3*, *Inpp5d*, *Ms4a6d* and *Spi1*/PU.1 itself, by searching data from a chromatin immunoprecipitation experiment against SPI1/PU.1 in mouse microglial-like BV-2 cells (Satoh *et al.*, 2014). This finding was supported by mining for regulatory features and *cis*-regulatory modules in the gene network expressed by microglia that respond to plaques using i-*cis*Target that uses a library of regulatory data (Imrichova *et al.*, 2015). Together, these findings suggest that several of the predicted and established Alzheimer’s disease risk genes may be regulated by SPI1/PU.1, which itself alters Alzheimer’s disease risk by coordinating a program of microglial-expressed genes (Huang *et al.*, 2017).

### Colocalization between Alzheimer’s disease-related loci and expression quantitative trait loci for gene *OAS1*

**Figure 2:**
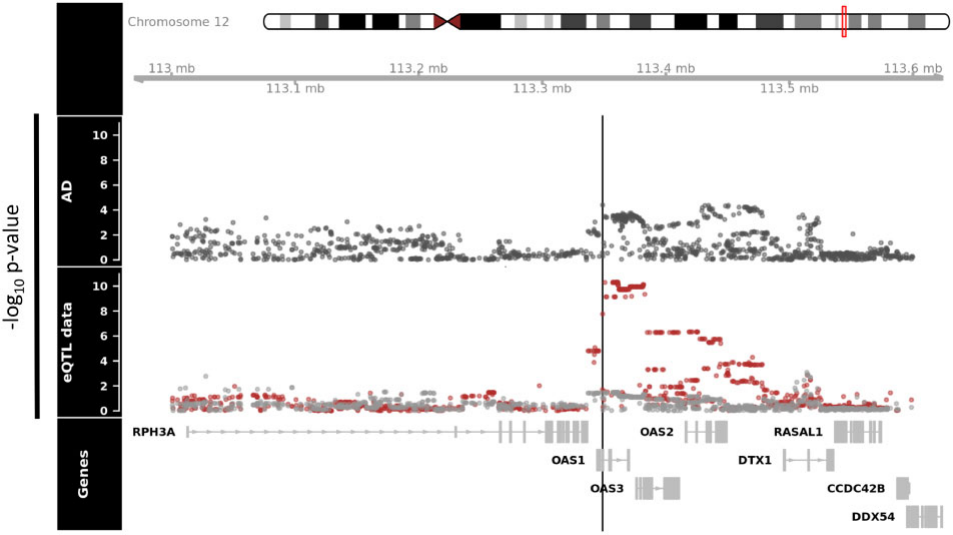
**Colocalization of Alzheimer’s disease GWAS loci with eQTLs derived from baseline and stimulated iPSC-derived macrophages.** Colocalization of Alzheimer’s disease loci and eQTLs targeting *OAS1* in baseline and stimulated states (interferon-γ and Salmonella, 18 and 5 h, respectively). In the eQTL panels, grey and red data points represent macrophages at baseline or stimulated with both interferon-γ and Salmonella, respectively. The eQTL data are from Alasoo *et al.* (2018). The best Alzheimer’s disease locus in *OAS1* from the IGAP data (Lambert *et al.*, 2013) is highlighted with the black line. Numerical results are reported in Supplementary Table 4.

Since most GWAS loci are thought to operate by regulating the expression of neighbouring genes (Bradshaw *et al.*, 2013; Griciuc *et al.*, 2013; Huang *et al.*, 2017), for each of the four potential Alzheimer’s disease-associated genes we performed a colocalization analysis to test the association between Alzheimer’s disease-related loci located within these genes and loci regulating the expression of these genes (eQTLs) (Giambartolomei *et al.*, 2014). eQTLs were obtained from two previously published datasets using baseline and stimulated human-derived monocytes and iPSC-derived macrophages (Kim-Hellmuth *et al.*, 2017; Alasoo *et al.*, 2018). In these studies, macrophages and monocytes were stimulated with various immunostimulants to activate distinct, well-characterised immune signalling pathways, including those broadly associated with bacterial and viral responses. Interestingly, we identified three colocalizations between Alzheimer’s disease loci and eQTLs regulating *OAS1* gene expression, all of which were identified in stimulated states, suggesting that this association is only active in certain environmental conditions (Fig. 2 and Supplementary Figs 8 and 9), in particular those designed to model monocyte/macrophage priming or more chronic inflammation.

## Discussion

A decade of GWAS projects for Alzheimer’s disease has provided key and initially surprising insights into the progression of late-onset Alzheimer’s disease, particularly the dependence on the innate immune system, with the identification of genes such as *TREM2* and *SPI1*/PU.1 (Guerreiro *et al.*, 2013; Jonsson *et al.*, 2013; Huang *et al.*, 2017; Sims *et al.*, 2017). The latest GWAS studies published during 2019 mark the largest of their kind for Alzheimer’s disease featuring 71 880 Alzheimer’s disease cases to identify 9 novel risk loci (Jansen *et al.*, 2019), and 35 274 clinically assessed Alzheimer’s disease cases to identify 5 novel risk loci from the updated IGAP study (Kunkle *et al.*, 2019). Despite all the risk genes that have been discovered by GWAS, they still do not account for all of the heritability of late-onset Alzheimer’s disease. Finding further risk genes will become increasingly difficult due to the sheer number of patients required and associated costs, as the remaining risk genes are likely to be of rare mutation frequency or lower effect size. Here, we describe a new approach to identify further risk genes by intersecting transcriptome data from a functional cellular response to rising amyloid with a gene-based statistical approach to identify genes significantly associated with Alzheimer’s disease from the updated IGAP project. We identify four further potential risk genes, *OAS1*, *LAPTM5*, *ITGAM*/CD11b and *LILRB4*, alongside confirming the importance of seven established GWAS hits *TREM2*, *ABI3*, *CD33*, *INPP5D, SPI1*/PU.1, *MS4A6D* and *GAL3ST4*. Together these new and established genes form a transcriptional network that is conserved in mice and humans, and so suggests that this sub-network of genes are regulated together, in part by the SPI1/PU.1 transcription factor, and may function together.

Surveying the literature on our genes of interest revealed that *OAS1* (2-prime, 5-prime oligoadenylate synthetase 1) is involved in the regulation of cytokine expression (Lee *et al.*, 2019). *OAS1* is induced by interferons (Donovan *et al.*, 2013), which supports our eQTL analysis showing that one of the best SNPs we identified for *OAS1* appears in a locus which acts as an eQTL in response to interferon-γ (IFNγ; Fig. 2 and Supplementary Figs 8 and 9). *OAS1* can additionally activate ribonuclease L, which degrades viral RNA and inhibits viral replication (Donovan *et al.*, 2013). Interferons are cytokines that are thought to trigger a key response to viral and other pathogens. In addition to the mouse orthologue of *OAS1* (*Oas1a*), a number of other genes involved in interferon signalling are also present in our co-expression network from amyloid-responsive microglia, including other *Oas* family members, *Ifit* members, and transcription factors such as *Irf7*, *Trp53* and the *Stat* family (Supplementary Tables 1 and 2). Recent studies have also shown that interferon-related genes are expressed in ageing control mice, and that the expression of interferon-related genes is further elevated in mouse models with amyloid pathology (Friedman *et al.*, 2018; Sala Frigerio *et al.*, 2019), leading to the identification of a population of ‘interferon response microglia’ (Sala Frigerio *et al.*, 2019). The role of *OAS1* and the other interferon-related genes in ageing animals and Alzheimer’s disease is not clear, they may be involved in limiting viral infections, recruiting immune cells to sites of damage and/or regulating cytokine production.

*LAPTM5* (lysosome-associated protein, transmembrane 5) is associated with amyloid deposition in transgenic mice (Nam *et al.*, 2018). *LILRB4* (leukocyte immunoglobulin-like receptor, subfamily B, member 4), orthologues have also been shown to be increased with amyloid deposition and specifically associated with amyloid plaques (Wirz *et al.*, 2013; Kamphuis *et al.*, 2016; Castillo *et al.*, 2017). A paralogue of *LILRB4*, named *LILRB2*, and its mouse orthologue *Pirb* have been shown to bind Aβ, and this interaction with Aβ in mice mediates synapse elimination, and deficits in synaptic plasticity and memory (Kim *et al.*, 2013). The functions of *LAPTM5* and *LILRB4* have not been well characterised, but are thought to suppress the activation of a variety of immune cells. *ITGAM*/CD11b (or CR3A), is a cell surface receptor involved in activation, migration and phagocytosis of immune cells, so much so that ITGAM/CD11b is used as a marker of activated microglia (Matsuoka *et al.*, 2001; Heneka *et al.*, 2013; Kamphuis *et al.*, 2016). *ITGAM*/CD11b was highlighted in recent genetic and functional analyses as likely being important for the progression of Alzheimer’s disease, whose expression was driven by SPI1/PU.1, and related to amyloid deposition in mice and humans (Zhang *et al.*, 2013; Hong *et al.*, 2016; Kamphuis *et al.*, 2016; Olmos-Alonso *et al.*, 2016; Huang *et al.*, 2017; Nam *et al.*, 2018). Most recently, inhibiting the interaction between the blood protein fibrinogen and ITGAM/CD11b reduced synaptic elimination and cognitive decline in a mouse model of Alzheimer’s disease (Merlini *et al.*, 2019), providing strong evidence that ITGAM/CD11b function contributes to disease development. Given the previous studies for *ITGAM*/CD11b, *LAPTM5* and *LILRB4*, it is tempting to speculate that they are involved in phagocytic processes involving synapses which are known to be reactivated during Alzheimer’s disease progression. More work is necessary to understand the molecular mechanisms of all four of these putative risk genes in the progression of Alzheimer’s disease.

It is also useful to consider how microglial proliferation in response to amyloid plaques relates to expression of the four putative risk genes. We have previously shown that microglial number is increased in these homozygous *APP/PSEN1* mice, by around 3.7-fold in the CA1 region of the hippocampus (Medawar *et al.*, 2019), and an elegant study by Srinivasan *et al.* (2016), delineates the difference between expression changes in bulk tissue versus the influence of increased microglial numbers in response to amyloid by cell sorting to analyse expression changes in purified microglia alone. The expression levels of our putative risk genes relative to expression in age-matched wild-type mice shows a range, with *Oas1a* showing the greatest relative expression (10.0-fold increase relative to wild-type), and *Itgam*/CD11b showing the lowest relative expression (2.3-fold increase), suggesting that these genes may fulfil different purposes in microglia in the presence of amyloid plaques. Genes showing higher relative transcript levels such as *Oas1a* and *Trem2* are likely to be directly up-regulated by microglia in response to amyloid, and may be promoting a protective response to amyloid e.g. as described by Lee *et al.* (2018). *Oas1a* shows increased expression in purified microglia from a number of different mouse models of Alzheimer’s disease, using the Myeloid Landscape datasets suggesting *Oas1a* is directly up-regulated by amyloid (http://research-pub.gene.com/BrainMyeloidLandscape/#; Friedman *et al.*, 2018). *Laptm5* and *Lilra5* relative expression are only significantly increased in homozygous *APP/PSEN1* mice when the plaque load starts to become heavy (8 months of age), suggesting direct regulation by amyloid only as the plaque load increases, implying a specific role for these genes in microglia at this stage. Instead, *Itgam*/CD11b shows a similar change in relative expression to the average relative transcript level of the entire immune network, and to the increase in microglia number, comparable to relative *Spi1*/PU.1 expression. This suggests that *Itgam*/CD11b and *Spi1*/PU.1 genes may play a role in regulating the change in microglia number in response to amyloid plaques, given the strong correlation between the expression of these genes and amyloid pathology.

The study by Huang *et al.* (2017) shows that a common SNP in the population delays onset of Alzheimer’s disease, purportedly via reduced expression of *SPI1*/PU.1. However, in our study we see a positive correlation between *Spi1*/PU.1 and candidate genes *Laptm5* and *Itgam*/CD11b, as well as established risk genes *Trem2* and *Abi3*, which all have binding sites in their promoters for SPI1/PU.1, suggesting that SPI1/PU.1 is a positive regulator of these genes in this mouse model where heavy amyloid load does not lead to tangles and neurodegeneration. This discrepancy may be due to differences in the increase in microglial number between mice and humans; our data suggest that *Spi1*/PU.1 may be regulating microglial number, and it is possible that the level of microglial proliferation that can be tolerated by mice and humans is different (particularly given the long course of Alzheimer’s disease in humans). Not all Alzheimer’s disease risk genes have SPI1/PU.1 binding sites; thus, while this core transcription factor plays a substantial role in the progression of disease, there are likely to be auxiliary, environment-dependent transcription factors that modify disease development. In future work, it would be good to complement the bulk RNA-seq analysis here with isolated microglia and single-cell work for microglia to determine how *Spi1*/PU.1 expression and the transcriptome is different for microglia proximal to plaques versus those away from plaques, and in different regions of the brain. In studies where microglia are isolated, the limitations associated with purifying microglia should be borne in mind, in that the procedure may alter some transcripts, and it is also important to consider the heterogeneity of microglia seen from single-cell work (Sala Frigerio *et al.*, 2019). Further work is required to understand how the putative risk genes respond to amyloid within microglia, both at the transcriptional level, and at the post-translational level. Notably, while there is evidence that these putative risk genes have been coincidentally linked with amyloid plaques, there is no published evidence to date that DNA variation in these genes in the human population is linked to risk for Alzheimer’s disease.

Our data also show that microglia respond differently to amyloid deposition versus tauopathy, with around 29% of transcripts in amyloid-responsive microglia showing a stronger correlation to amyloid pathology. A recent study also presents related data, identifying a co-expression module within microglia that respond more robustly to amyloid pathology compared to tauopathy (Sierksma *et al.*, 2019). In both studies, established and putative Alzheimer’s disease risk genes are more strongly enriched in the amyloid-responsive microglia compared to tauopathy-responsive microglia. These data collectively provide compelling evidence that the microglial response to amyloid pathology determines whether the disease progresses to neurodegeneration and cognitive problems. Further work is required to understand how the microglial response to tauopathy is different, and why mouse models with heavy amyloid plaque loads do not lead to tau tangles and neurodegeneration. It may be that other triggers, in addition to amyloid deposition, are required to push microglia to a state that permits amyloid-dependent tau pathology, such as blood–brain barrier breakdown or priming of the immune system by exposure to environmental pathogens. Alternatively, it may be due to microglial genes expressed more abundantly in human microglia compared to mouse.

This work focuses on the commonality between mice and humans, specifically how expression of mouse microglial genes overlap with human genes showing DNA variation associated with Alzheimer’s disease. It is worthwhile to bear in mind that a number of important studies have compared gene expression in microglia from mice and humans, and while they have shown a significant overlap between the transcriptomes of the two species, they have also seen a number of genes are expressed selectively more abundantly in human microglia (Miller *et al.*, 2010; Galatro *et al.*, 2017; Gosselin *et al.*, 2017). Our four putative risk genes, *OAS1*, *LAPTM5*, *LILRB4* and *ITGAM*/CD11b are expressed abundantly in the human microglia (Galatro *et al.*, 2017; Gosselin *et al.*, 2017), and more generally there is a substantial overlap in human orthologues expressed by the mouse amyloid-responsive microglia and the transcripts expressed abundantly by human microglia from Galatro *et al.* (2017) and Gosselin *et al.* (2017). Genes expressed more abundantly in human microglia and not present in our mouse microglial network are given in Supplementary Table 5. Thus, in future studies it is important to select the appropriate model for the study of specific microglia genes.

The importance of this work is 2-fold. First, by identifying more genetic loci involved in amyloid deposition, we derive a more complete insight into the cellular processes and molecular mechanisms underlying the disease. In this regard, this work is complementary to that of Huang *et al.* (2017), showing that microglial SPI1/PU.1-driven transcription is a common feature of many Alzheimer’s disease loci. These findings are also consistent with previous work on *Trem2* (Wang *et al.*, 2015; Keren-Shaul *et al.*, 2017; Mazaheri *et al.*, 2017; Cheng-Hathaway *et al.*, 2018; Lee *et al.*, 2018), and *CD33* (Bradshaw *et al.*, 2013; Griciuc *et al.*, 2013), suggesting these risk genes are crucial in controlling the microglial response to amyloid-induced damage. Understanding the mechanisms of function of TREM2 and the sub-network of genes expressed by amyloid-responsive microglia identified here may be useful to leverage therapeutic opportunities. Second, and perhaps of greater importance, this work implies that, overall, how well an individual responds to amyloid deposition at the cellular and gene expression level plays a part in determining one’s risk of disease, and understanding the genes that control this may be used to predict the chances of developing Alzheimer’s disease and to develop preventative or disease-delaying treatments before irreversible neurodegeneration sets in.

## Supplementary Material

fcz022_Supplementary_Data

## Abbreviations

Aβ =: amyloid beta
APP =: amyloid precursor protein
eQTL =: expression quantitative trait loci
GWAS =: genome-wide association studies IFNγ, interferon-γ
IGAP: International Genomics of Alzheimer’s Projects; SNP, single nucleotide polymorphism
