# Supplementary material for: Genetic variability in response to amyloid beta deposition influences Alzheimer’s disease risk
Source: Brain Commun. 2019 Oct 10;1(1):fcz022. doi: 10.1093/braincomms/fcz022 (PMC7145452; doi:10.1093/braincomms/fcz022)
Supplement: fcz022_Supplementary_Data [file fcz022_supplementary_data.zip › Supplementary material.pdf]

Supplementary material

Additional materials and methods

Mouse models of Alzheimer's disease

The RNA samples used for this study were from the same mice we used previously, described in detail in Matarin *et al.*, (2015), therefore no further mice were bred for this study. Briefly, the hippocampi of male mice at 2, 4, 8, or 18 months were used. At least three mice were used from each of the following five transgenic lines (except for the TAS10-mice at 4 months, and tau-mice at 18 months, where only two mice were available), plus at least seven littermate controls from the original parental lines. The five mouse lines used were as follows:

TAS10-mice: human *APP* (K670N/M671L); hemizygous on *Thy1* promoter (Richardson *et al.*, 2003),

TPM-mice: human *PSEN1* (M146V); hemizygous on *Thy1* promoter (Howlett *et al.*, 2004),

HET-TASTPM-mice: hemizygous for both of the *APP* and *PSEN1* transgenes (Medawar *et al.*, 2019),

HO-TASTPM-mice: as above but both transgenes bred to homozygosity (Medawar *et al.*, 2019),

Tau-mice: human microtubule-associated protein tau (*MAPT*; P301L),

hemizygous on *Camk2a* promoter (Joel *et al.*, 2019),

WT-mice: age-matched wild-type mice of the same C57Bl/6J background.

Mouse transcriptome work

The quality and concentration of the total RNA from the Matarin *et al.*, (2015) cohort was assessed using capillary electrophoresis of each sample. For this new study, RNA-seq library preparation and sequencing was performed by Eurofins Genomics (Ebersberg, Germany). RNA strand-specific libraries were created using commercially available kits according to the manufacturer's instructions (TruSeq Stranded mRNA Library Preparation Kit, Illumina). In brief, poly(A)-RNA was extracted from total RNA using an oligo(dT)-bead based method. After fragmentation of the mRNA, first-strand and dUTP-based second strand synthesis was carried out, followed by end-repair, A-tailing, ligation of the indexed Illumina adapter and digestion of the dUTP-strand. Size selection was performed using a bead-based method. After PCR amplification, the resulting fragments were purified, pooled, quantified and used for cluster generation. For sequencing, pooled libraries were loaded on the cBot (Illumina) and cluster generation was performed according the manufacturer's instructions. Paired-end sequencing using 100 bp read length (multiplex 12 samples per lane - 28M reads) was performed on a HiSeq2500 (HiSeq Control Software 2.2.58) using HiSeq Flow Cell v4 and TruSeq SBS Kit v4. For processing of raw data RTA version 1.18.64 and bcl2fastq-1.8.4 was used to generate FASTQ files. Adaptors and low quality base pairs were removed from FASTQ files using Trim Galore (Babraham Bioinformatics). Transcripts were quantified with Salmon (Patro *et al.*, 2017), using gene annotation from ENSEMBL GRCm38. Salmon was used because it incorporates GC correction and accounts for fragment positional bias. To obtain gene level quantification from the transcripts, and correct for average transcript length and library size, expressed as transcripts per million (TPM), the tximport R package was used (Soneson *et al.*, 2015). TPM values were

log_2 transformed, and genes were considered expressed when log_2 TPM values displayed a mean >1·5 for a given gene, when gene TPM values were averaged for each genotype at each age (resulting in a total of 18,562 genes expressed).

Weighted gene co-expression network analyses (WGCNAs) were performed as described in Matarin *et al.*, (2015), using the recommended parameters from the original analysis developers (Zhang and Horvath, 2005; Horvath *et al.*, 2006; Oldham *et al.*, 2006; Langfelder and Horvath, 2008). R code and tutorials available from: https://horvath.genetics.ucla.edu/html/CoexpressionNetwork/Rpackages/WGCNA/index.html). Co-expression networks were built using the WGCNA package in R. Genes with variable expression patterns (coefficient of variation >5% for wild-type, amyloid and tau mice) from log_2 TPM values were selected for network analyses resulting in 13,536 genes for network analyses. The module of genes with the highest significant correlation of the module eigengene with amyloid or tau pathological features was selected for analysis (amyloid, correlation 0·94, p = $3e^{-41}$; tau, correlation 0·82, p = $4e^{-12}$). The gene module with the highest correlation to both amyloid and tau pathological features contained genes from the innate immune system. The topological overlap measure (TOM) connectivity values were used to plot the network diagrams (TOM > 0·39 for amyloid-associated module, and TOM > 0·36 for tau-associated module, to plot a similar number of genes, approximately the top 150 genes with the highest connections per module). Hub genes were considered to be those with at least 15 connections to other genes as Matarin *et al.*, (2015), and Miller *et al.,* (2010).

Human sample co-expression network construction and annotation

Co-expression networks were generated from RNA-seq based gene expression profiling of 635 pre-frontal cortex samples from the ROS/MAP project (Bennett *et al.*, 2012a; Bennett *et al.*, 2012b; De Jager *et al.*, 2018). The cognitive decline reported in the original studies cited above was used as a covariate to construct four networks: all samples network, not AD, probable AD and AD. WGCNA (Langfelder and Horvath, 2008) was used with an optimization for constructing more biologically meaningful co-expression networks (Botia *et al.*, 2017). Gene expression was $\log_2$ transformed previous to any data analysis task. Then batch effects were corrected using ComBAT (Johnson *et al.*, 2007), unknown hidden effect covariates with SVA were obtained (Leek and Storey, 2007), and the residuals obtained were used by regressing the gene expression with SVA covariates, age and gender. Then the network modules were annotated for enrichment of Gene Ontology, REACTOME (Fabregat *et al.*, 2018), and KEGG (Kanehisa *et al.*, 2016) pathways using gProfileR (Reimand *et al.*, 2007).

Colocalization with monocyte eQTL datasets

Coloc (version 3.1, see below for Software and algorithms) was applied to test for colocalization between AD loci surrounding the four novel risk genes (*OAS1*, *LAPTM5*, *ITGAM*, and *LILRB4*) and eQTLs (Giambartolomei *et al.*, 2014). While no microglial eQTL datasets exist to date, eQTL analyses have been performed using monocytes and iPSC-derived macrophages (at rest and stimulated with various immunostimulants, such as IFN-γ) (Kim-Hellmuth *et al.*, 2017; Alasoo *et al.*, 2018).

Coloc was run using default parameters and priors on all SNPs that: 1) had eQTLs tagging one of the four novel genes (this included all tested SNP-gene associations, including non-significant eQTLs); and 2) had overlapping SNPs in the AD GWAS. All loci were excluded for which PP3 + PP4 < 0·8, to exclude loci which were underpowered to detect colocalization. Loci with PP4/PP3 ≥ 2 were considered colocalized due to a single shared causal variant (PP4), as opposed to two distinct causal variants (PP3).

Software and algorithms

Mouseac, this paper and Matarin *et al.* (2015): www.mouseac.org

WGCNA (Langfelder and Horvath, 2008): https://horvath.genetics.ucla.edu/html/CoexpressionNetwork/Rpackages/WGCNA/index.html (accessed September 2018)

Braineac (Ramasamy *et al.*, 2014): www.braineac.org (accessed September 2018)

1,000 genomes (Genomes Project *et al.*, 2015): www.1000genomes.org and http://www.internationalgenome.org (accessed September 2018)

MAGMA (de Leeuw *et al.*, 2015): www.ctg.cncr.nl/software/magma (accessed May 2019)

Coloc, version 3.1, (Giambartolomei *et al.*, 2014): https://github.com/chr1swallace/coloc (accessed September 2018)

ROS/MAP (Bennett *et al.*, 2012a; Bennett *et al.*, 2012b; De Jager *et al.*, 2018): https://www.synapse.org/#!Synapse:syn3219045 (accessed September 2018)

i-CisTarget (Imrichova *et al.*, 2015): https://gbiomed.kuleuven.be/apps/lcb/i-cisTarget (accessed September 2018)

GTEx V6 gene expression (Consortium GT, 2015): https://gtexportal.org/home (accessed September 2018)

Coexp (Botia *et al.*, 2017): https://github.com/juanbot/CoExpNets (accessed September 2018)

Myeloid landscape datasets (Friedman *et al.*, 2018): http://research-pub.gene.com/BrainMyeloidLandscape/# (accessed June 2019)

References

Alasoo K, Rodrigues J, Mukhopadhyay S, Knights AJ, Mann AL, Kundu K*, et al.* Shared genetic effects on chromatin and gene expression indicate a role for enhancer priming in immune response. Nat Genet 2018; 50(3): 424-31.

Bennett DA, Schneider JA, Arvanitakis Z, Wilson RS. Overview and findings from the religious orders study. Curr Alzheimer Res 2012a; 9(6): 628-45.

Bennett DA, Schneider JA, Buchman AS, Barnes LL, Boyle PA, Wilson RS. Overview and findings from the rush Memory and Aging Project. Curr Alzheimer Res 2012b; 9(6): 646-63.

Botia JA, Vandrovcova J, Forabosco P, Guelfi S, D'Sa K, United Kingdom Brain Expression C*, et al.* An additional k-means clustering step improves the biological features of WGCNA gene co-expression networks. BMC Syst Biol 2017; 11(1): 47.

Consortium GT. Human genomics. The Genotype-Tissue Expression (GTEx) pilot analysis: multitissue gene regulation in humans. Science 2015; 348(6235): 648-60.

De Jager PL, Ma Y, McCabe C, Xu J, Vardarajan BN, Felsky D*, et al.* A multi-omic atlas of the human frontal cortex for aging and Alzheimer's disease research. Sci Data 2018; 5: 180142.

de Leeuw CA, Mooij JM, Heskes T, Posthuma D. MAGMA: generalized gene-set analysis of GWAS data. PLoS Comput Biol 2015; 11(4): e1004219.

Fabregat A, Jupe S, Matthews L, Sidiropoulos K, Gillespie M, Garapati P*, et al.* The Reactome Pathway Knowledgebase. Nucleic acids research 2018; 46(D1): D649-D55.

Friedman BA, Srinivasan K, Ayalon G, Meilandt WJ, Lin H, Huntley MA*, et al.* Diverse Brain Myeloid Expression Profiles Reveal Distinct Microglial Activation States and Aspects of Alzheimer's Disease Not Evident in Mouse Models. Cell Rep 2018; 22(3): 832-47.

Galatro TF, Holtman IR, Lerario AM, Vainchtein ID, Brouwer N, Sola PR, *et al.* Transcriptomic analysis of purified human cortical microglia reveals age-associated changes. Nat Neurosci 2017; 20(8): 1162-71.

Genomes Project C, Auton A, Brooks LD, Durbin RM, Garrison EP, Kang HM*, et al.* A global reference for human genetic variation. Nature 2015; 526(7571): 68-74.

Giambartolomei C, Vukcevic D, Schadt EE, Franke L, Hingorani AD, Wallace C*, et al.* Bayesian test for colocalisation between pairs of genetic association studies using summary statistics. PLoS Genet 2014; 10(5): e1004383.

Gosselin D, Skola D, Coufal NG, Holtman IR, Schlachetzki JCM, Sajti E, *et al.* An environment-dependent transcriptional network specifies human microglia identity. Science 2017; 356(6344).

Horvath S, Zhang B, Carlson M, Lu KV, Zhu S, Felciano RM*, et al.* Analysis of oncogenic signaling networks in glioblastoma identifies ASPM as a molecular target. Proc Natl Acad Sci U S A 2006; 103(46): 17402-7.

Howlett DR, Richardson JC, Austin A, Parsons AA, Bate ST, Davies DC*, et al.* Cognitive correlates of Abeta deposition in male and female mice bearing amyloid precursor protein and presenilin-1 mutant transgenes. Brain Res 2004; 1017(1-2): 130-6.

Imrichova H, Hulselmans G, Atak ZK, Potier D, Aerts S. i-cisTarget 2015 update: generalized cis-regulatory enrichment analysis in human, mouse and fly. Nucleic acids research 2015; 43(W1): W57-64.

Joel Z, Izquierdo P, Salih DA, Richardson JC, Cummings DM, Edwards FA. Improving Mouse Models for Dementia. Are All the Effects in Tau Mouse Models Due to Overexpression? Cold Spring Harb Symp Quant Biol 2019; LXXXIII.

Johnson WE, Li C, Rabinovic A. Adjusting batch effects in microarray expression data using empirical Bayes methods. Biostatistics 2007; 8(1): 118-27.

Kanehisa M, Sato Y, Kawashima M, Furumichi M, Tanabe M. KEGG as a reference resource for gene and protein annotation. Nucleic acids research 2016; 44(D1): D457-62.

Kim-Hellmuth S, Bechheim M, Putz B, Mohammadi P, Nedelec Y, Giangreco N*, et al.* Genetic regulatory effects modified by immune activation contribute to autoimmune disease associations. Nat Commun 2017; 8(1): 266.

Kunkle BW, Grenier-Boley B, Sims R, Bis JC, Damotte V, Naj AC*, et al.* Genetic meta-analysis of diagnosed Alzheimer's disease identifies new risk loci and implicates Abeta, tau, immunity and lipid processing. Nat Genet 2019; 51(3): 414-30.

Lambert JC, Ibrahim-Verbaas CA, Harold D, Naj AC, Sims R, Bellenguez C*, et al.* Meta-analysis of 74,046 individuals identifies 11 new susceptibility loci for Alzheimer's disease. Nat Genet 2013; 45(12): 1452-8.

Langfelder P, Horvath S. WGCNA: an R package for weighted correlation network analysis. BMCBioinformatics 2008; 9: 559.

Leek JT, Storey JD. Capturing heterogeneity in gene expression studies by surrogate variable analysis. PLoS Genet 2007; 3(9): 1724-35.

Matarin M, Salih DA, Yasvoina M, Cummings DM, Guelfi S, Liu W*, et al.* A genome-wide gene-expression analysis and database in transgenic mice during development of amyloid or tau pathology. Cell Rep 2015; 10(4): 633-44.

Medawar E, Benway TA, Liu W, Hanan TA, Haslehurst P, James OT*, et al.* Effects of rising amyloidbeta levels on hippocampal synaptic transmission, microglial response and cognition in APPSwe/PSEN1M146V transgenic mice. EBioMedicine 2019; 39: 422-35.

Miller JA, Horvath S, Geschwind DH. Divergence of human and mouse brain transcriptome highlights Alzheimer disease pathways. Proc Natl Acad Sci U S A 2010; 107(28): 12698-703.

Oldham MC, Horvath S, Geschwind DH. Conservation and evolution of gene coexpression networks in human and chimpanzee brains. Proc Natl Acad Sci U S A 2006; 103(47): 17973-8.

Patro R, Duggal G, Love MI, Irizarry RA, Kingsford C. Salmon provides fast and bias-aware quantification of transcript expression. Nat Methods 2017; 14(4): 417-9.

Ramasamy A, Trabzuni D, Guelfi S, Varghese V, Smith C, Walker R*, et al.* Genetic variability in the regulation of gene expression in ten regions of the human brain. Nat Neurosci 2014; 17(10): 1418-28.

Reimand J, Kull M, Peterson H, Hansen J, Vilo J. g:Profiler--a web-based toolset for functional profiling of gene lists from large-scale experiments. Nucleic acids research 2007; 35(Web Server issue): W193-200.

Richardson JC, Kendal CE, Anderson R, Priest F, Gower E, Soden P*, et al.* Ultrastructural and behavioural changes precede amyloid deposition in a transgenic model of Alzheimer's disease. Neuroscience 2003; 122(1): 213-28.

Soneson C, Love MI, Robinson MD. Differential analyses for RNA-seq: transcript-level estimates improve gene-level inferences. F1000Res 2015; 4: 1521.

Zhang B, Horvath S. A general framework for weighted gene co-expression network analysis. Stat Appl Genet Mol Biol 2005; 4: Article17.

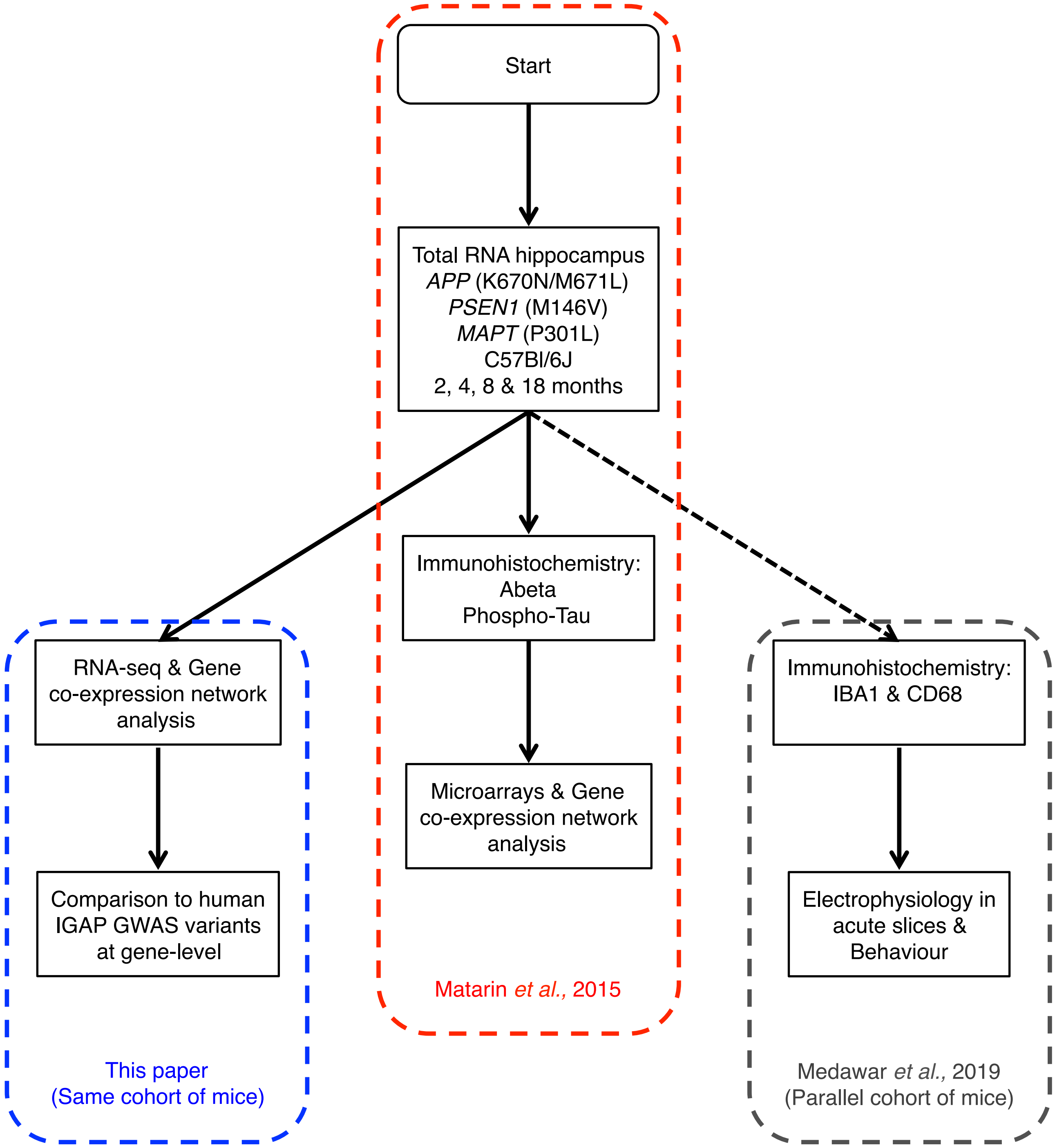

Supplementary Figure 1

Supplementary Figure 1 How the RNA-seq, gene co-expression analysis and then comparison of the genes in the mouse microglial co-expression module to human IGAP GWAS variants at the gene-level relate to other datasets we have published

A cohort of *APP* (K670N/M671L)/*PSEN1* (M146V) and tau (P301L) transgenic mice were bred for Matarin *et al.,* (2015), where the amyloid and tau pathology were assessed by immunohistochemistry, and microarrays were performed to identify gene expression changes and co-expression networks expressed by microglia associated with pathology. Parallel cohorts of mice from the *APP/PSEN1* lines (represented by the dashed arrow) were then used to count the increase in microglial number and activity in the CA1 of the hippocampus, alongside electrophysiological deficits in synaptic plasticity of acute hippocampal slices and mild changes to behaviour (Medawar *et al.,* 2019). For this new study, we performed RNA-seq using the total RNA from the hippocampus of the exact same mice used for study by Matarin *et al.*, (2015), and using gene co-expression network analyses we identified a higher resolution network expressed by amyloid-responsive or tau-responsive microglia. In this study we also statistically compare the genes present in the mouse microglial networks using human gene-level sequence variants associated with Alzheimer's disease as assessed in the IGAP GWAS (Kunkle *et al.,* 2019).

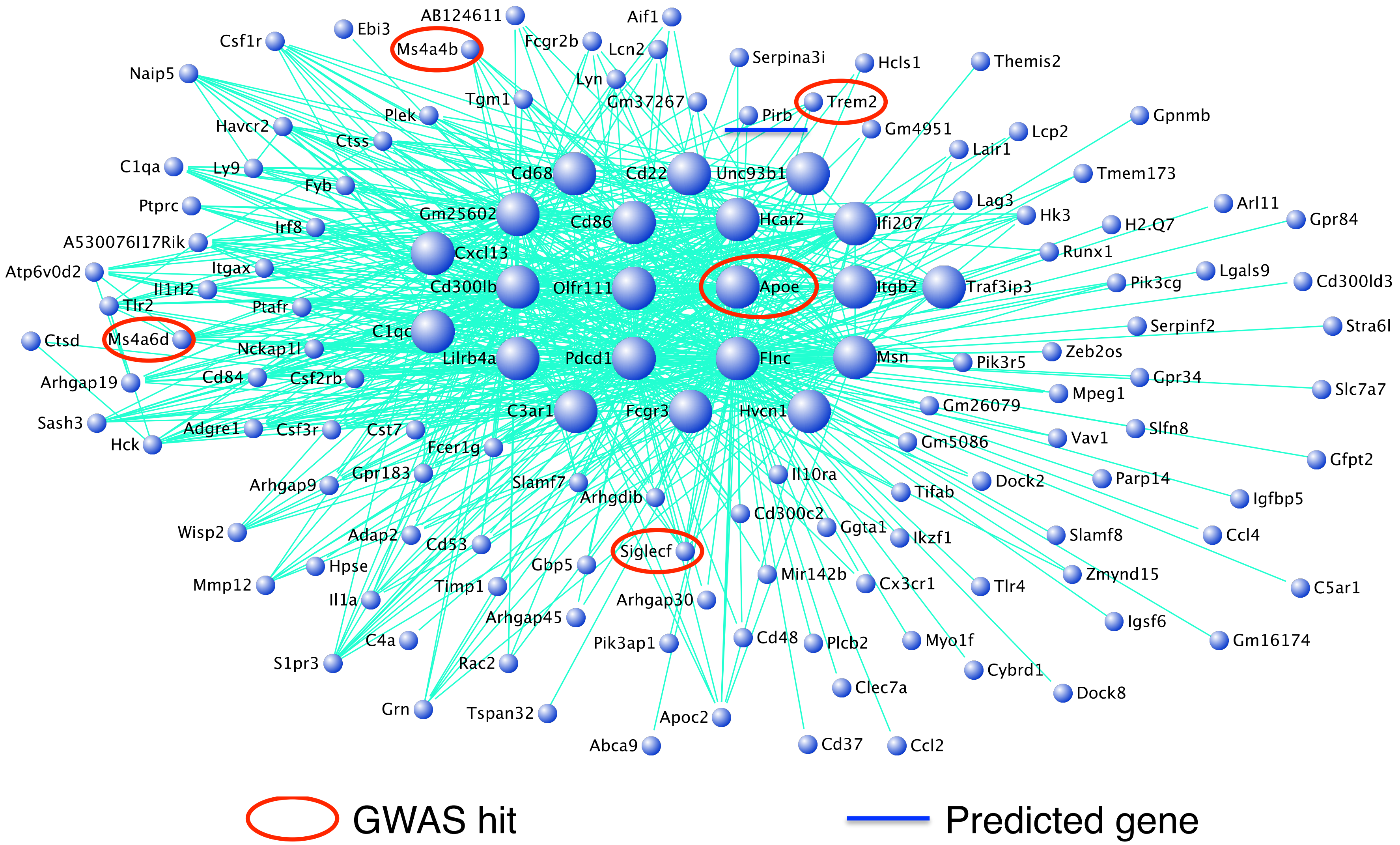

Supplementary Figure 2

Supplementary Figure 2 An innate immune network of genes expressed by microglia that respond to tau pathology, featuring some orthologues of established GWAS genes associated with Alzheimer's disease, is different to the immune network expressed by microglia responding to amyloid

Network plot using VisANT reveals key drivers of an innate immune module from RNA-seq derived gene expression from the hippocampus of wild-type and tau mice. Red circles show orthologues of established GWAS genes associated with Alzheimer's disease including *Trem2*, *Apoe*, *Ms4a6d* and *Ms4a4b*. Blue underline shows the single gene orthologue, *Pirb*, predicted to confer increased risk of Alzheimer's disease by overlapping gene expression data in microglia that respond to tau pathology in transgenic mice with individual human genes significantly associated with Alzheimer's disease by analyzing combinations of adjacent SNPs bounded by the coding regions of genes (compare to Fig. 1). Larger blue spheres represent 'hub' genes, those showing the greatest number of connections to other genes in the network, and include *Apoe*, *Cd68*, *C1qc* and *Lilrb4a*, which are likely to play important roles in driving microglial function in response to tau pathology.

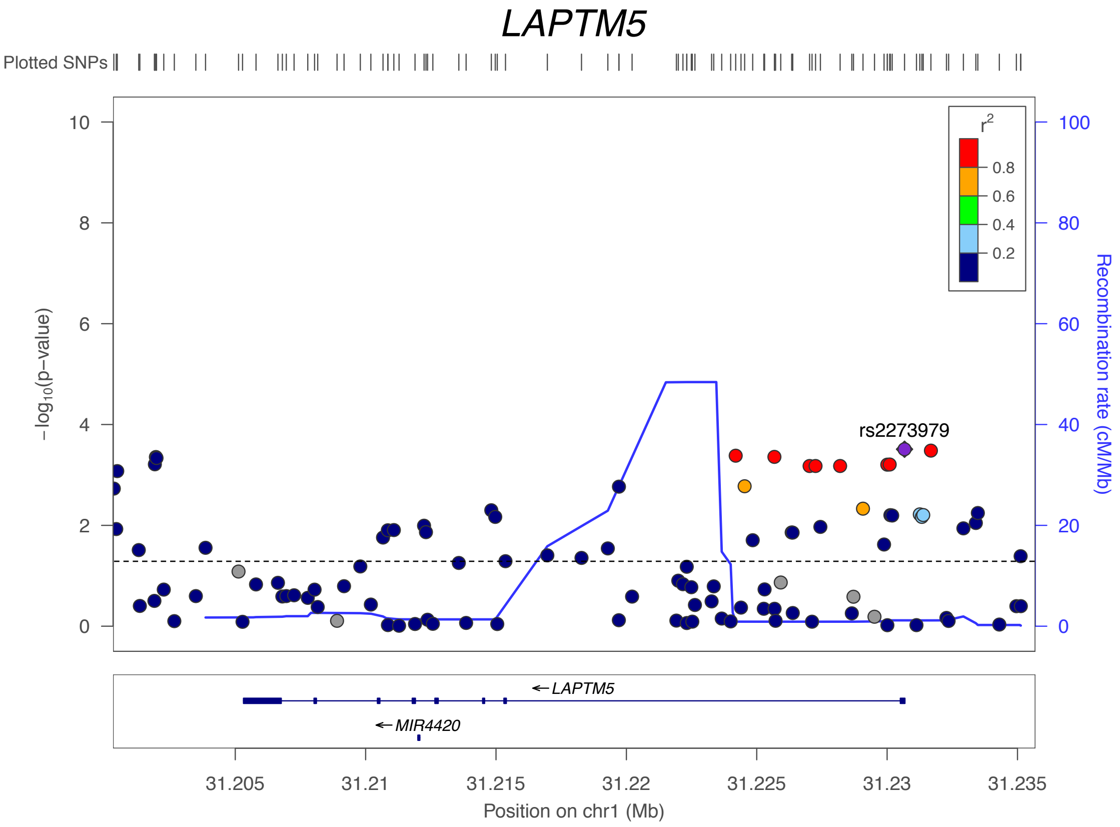

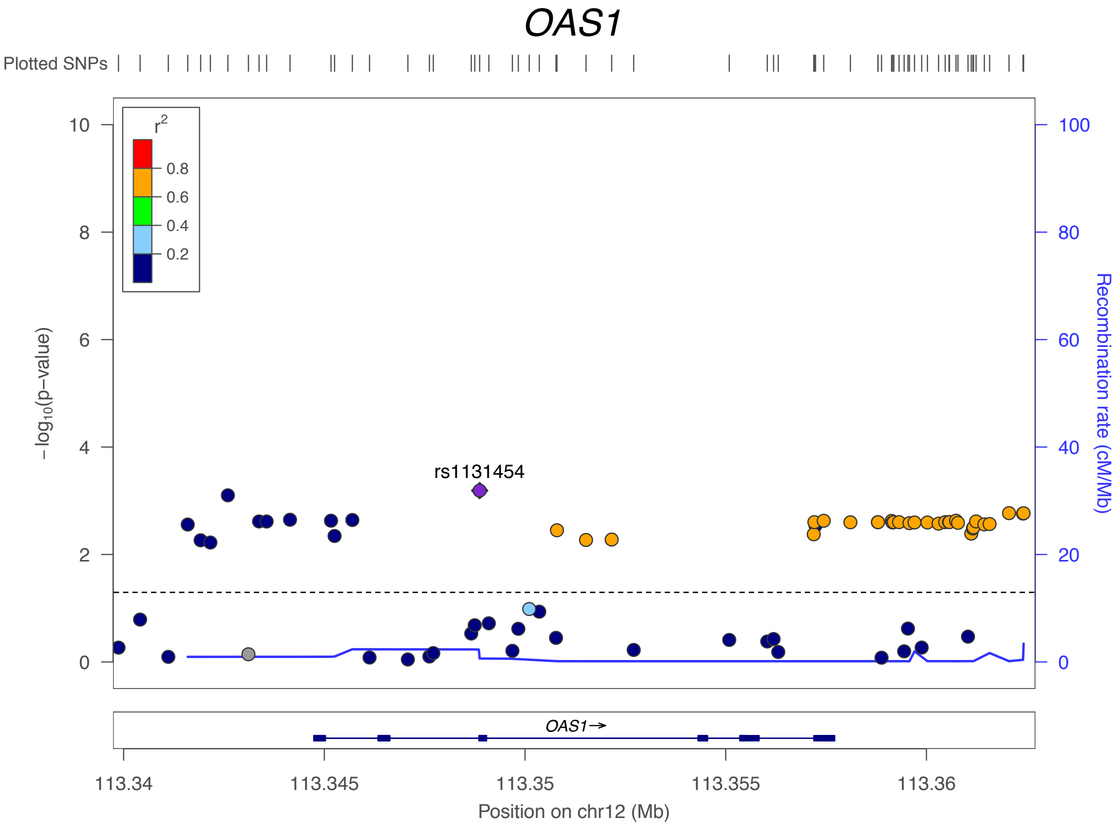

Supplementary Figure 3

Supplementary Figure 3 Regional plots for the *LAPTM5* and *OAS1* loci

The SNP positions are provided from Build 37, Assembly Hg19, and illustrated using LocusZoom software. The left y-axis gives the significance of each single SNP associated with Alzheimer's disease from IGAP, and the right y-axis gives the recombination rate along the gene where a higher recombination rate indicates greater independence between SNPs. The SNP with the most significant p-value for *LAPTM5* was rs7549164, and for *OAS1* was rs4766676 according to Kunkle *et al.*, (2019), but not available in the data used by the LocusZoom software for LD estimation. Dashed line illustrates p-value = 0.05.

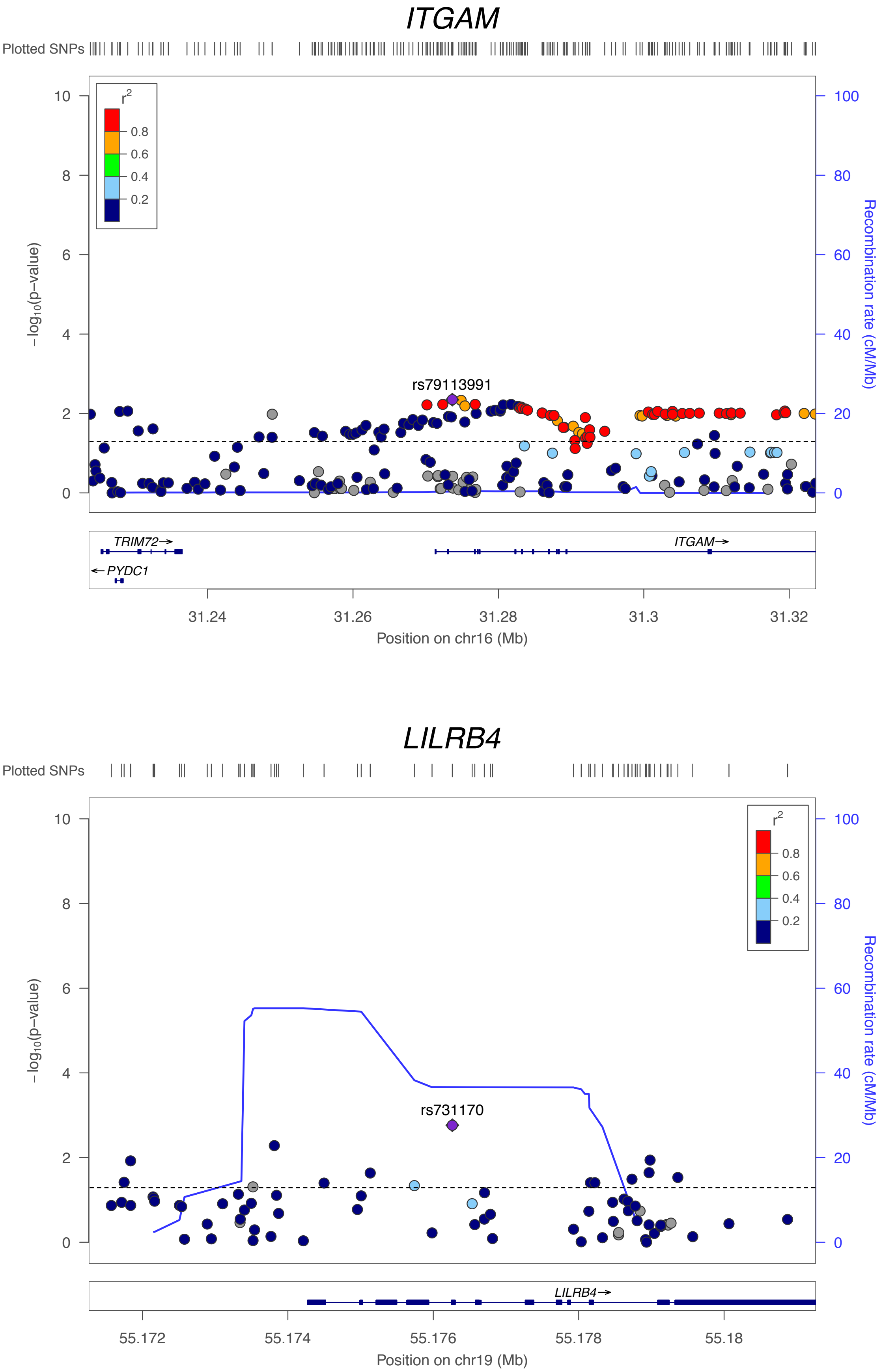

Supplementary Figure 4

Supplementary Figure 4 Regional plots for the *ITGAM* and *LILRB4* loci

The SNP positions are provided from Build 37, Assembly Hg19, and illustrated using LocusZoom software. The left y-axis gives the significance of each single SNP associated with Alzheimer's disease from IGAP, and the right y-axis gives the recombination rate along the gene where a higher recombination rate indicates greater independence between SNPs. The SNP with the most significant p-value within each gene is labelled, for *ITGAM* rs79113991, and for *LILRB4* rs731170 according to Kunkle *et al.*, (2019). Dashed line illustrates p-value = 0.05.

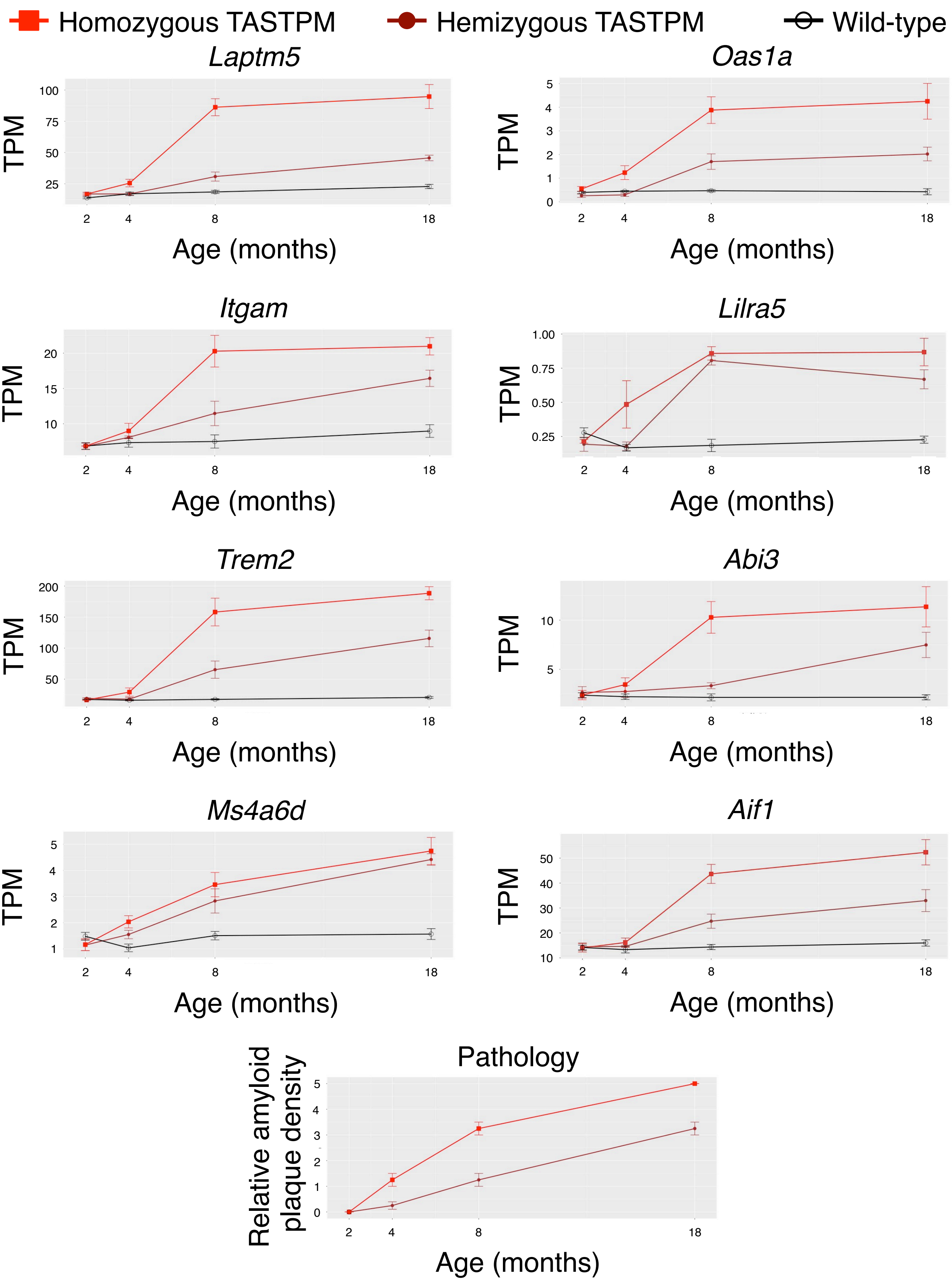

Supplementary Figure 5

Supplementary Figure 5 Expression of the orthologues of the four genes predicted to confer increased risk of Alzheimer's disease alongside established GWAS risk genes, *Trem2*, *Abi3* and *Ms4a6d*, and a reference *Aif1* in *APP/PSEN1* mice in response to amyloid deposition. Gene expression is presented from the RNA-seq as transcripts per million, TPM.

A

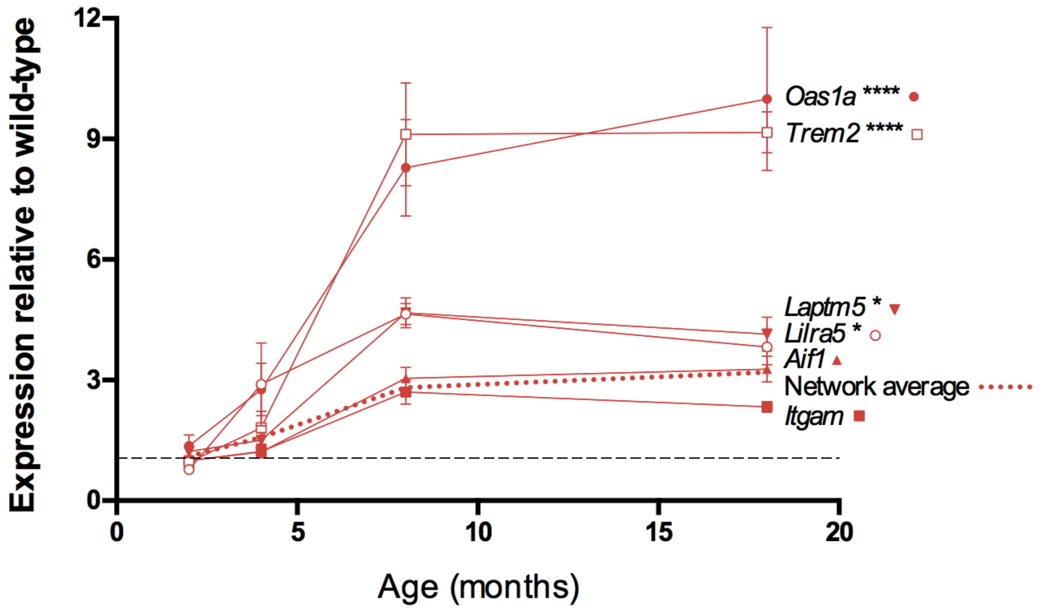

B

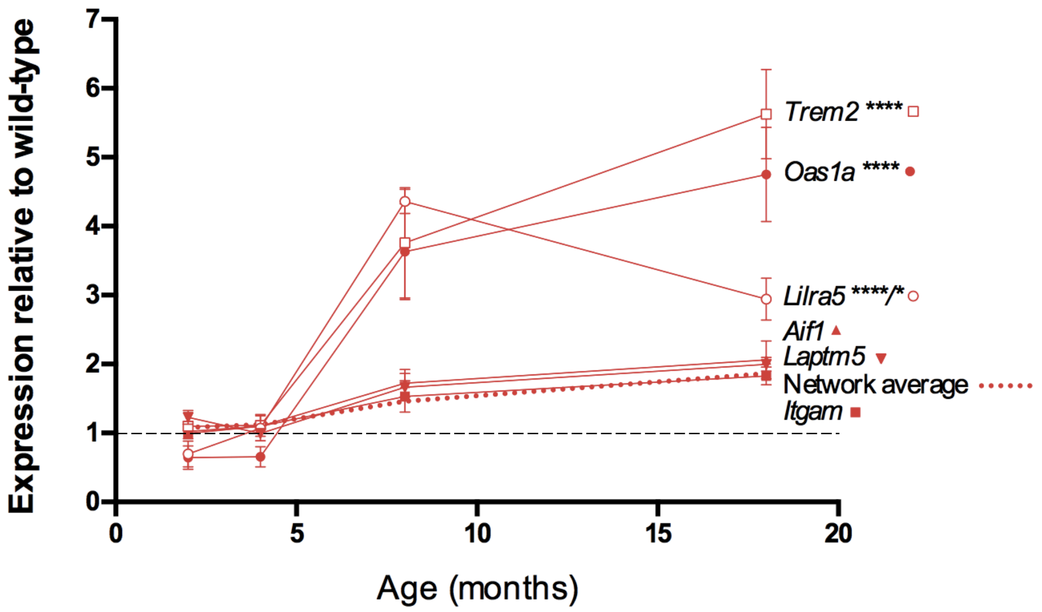

Supplementary Figure 6

Supplementary Figure 6 Relative expression of the orthologues of the four genes predicted to confer increased risk of Alzheimer's disease alongside the established GWAS risk gene *Trem2* and a reference *Aif1* in *APP/PSEN1* mice. (A) The expression of the genes in the homozygous *APP/PSEN1* mice is shown relative to age-matched wild-type mice. The dotted red line represents the average expression of 1,584 genes in the innate immune network (Supplementary Table 1) in homozygous *APP/PSEN1* mice relative to age-matched wild-type mice (dashed black line = 1.0). N = 3-4 homozygous *APP/PSEN1* and N = 7-8 wild-type mice per age group. Data shown as mean ± SEM. Two-way ANOVA with significant main effects of genotype ($p < 0.0001$), age ($p < 0.0001$) and significant interaction ($p < 0.0001$). Dunnett's multiple comparisons tests were used to test pairwise significance between each gene and the average relative expression of all genes in the innate immune network: *Oas1a* and *Trem2* were significant at 8- and 18-months of age compared to average relative expression of all genes in network; **** $p<0.0001$. *Laptm5* and *Lilra5* were significant only at 8-months of age compared to average relative expression of all genes in network; * $p<0.05$.

(B) The expression of the genes in the hemizygous *APP/PSEN1* mice is shown relative to age-matched wild-type mice. The dotted red line represents the average expression of 1,584 genes in the innate immune network (Supplementary Table 1) in hemizygous *APP/PSEN1* mice relative to age-matched wild-type mice (dashed black line = 1.0). N = 4 hemizygous *APP/PSEN1* and N = 7-8 wild-type mice per age group. Data shown as mean ± SEM. Two-way ANOVA with significant main effects of genotype ($p < 0.0001$), age ($p < 0.0001$) and significant interaction ($p < 0.0001$). Dunnett's multiple comparisons tests were used to test pairwise significance between each gene and the average relative expression of all genes in the innate immune

network: *Oas1a* and *Trem2* were significant at 8- and 18-months of age compared to average relative expression of all genes in network; **** $p<0.0001$. *Lilra5* was significant at 8-months of age (**** $p<0.0001$), and significant at 18-months of age (* $p<0.05$), compared to age-matched average relative expression of all genes in network.

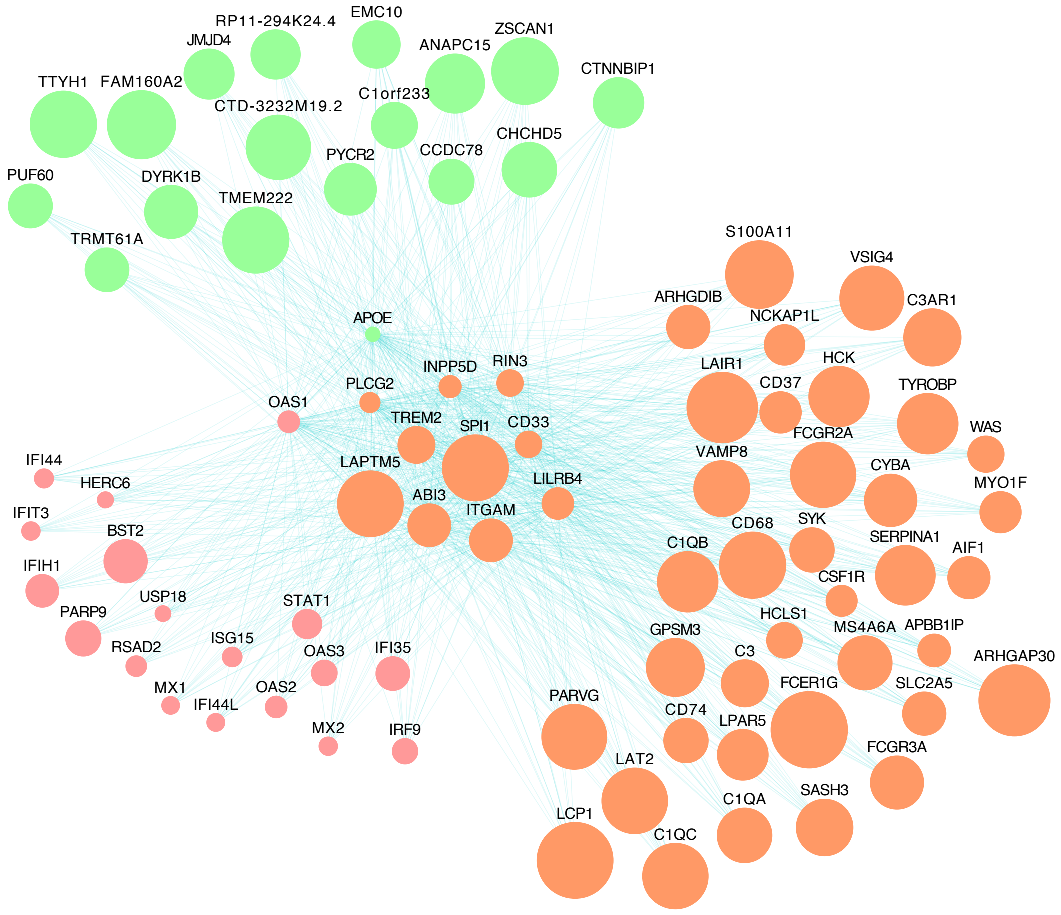

Supplementary Figure 7

Supplementary Figure 7 Co-expression network of *LAPTM5*, *ITGAM*, *LILRB4* and *OAS1* in the human ROS/MAP samples

The genes *LAPTM5*, *ITGAM*, *LILRB4*, *OAS1*, *TREM2*, *ABI3*, *CD33*, *SPI1*, *INPP5D*, *PLCG2*, *RIN3* and *APOE* were used as seeds, and for each seed gene, sequentially, the genes most connected to it were added (based on TOM values). This allows visualization of the connectivity context around specific genes that are not necessarily clustering together (i.e. they belong to different network modules), but likely cross-talk and interact. The colours indicate that the genes belong to three modules. Annotating the modules for enrichment with Gene Ontology, REACTOME and KEGG reveals that the Tan module relates to innate immune system function, the Pink module relates to interferon signaling, and the Green module relates to metabolic processes. The size of the sphere reflects how likely the gene is to act as a hub gene.

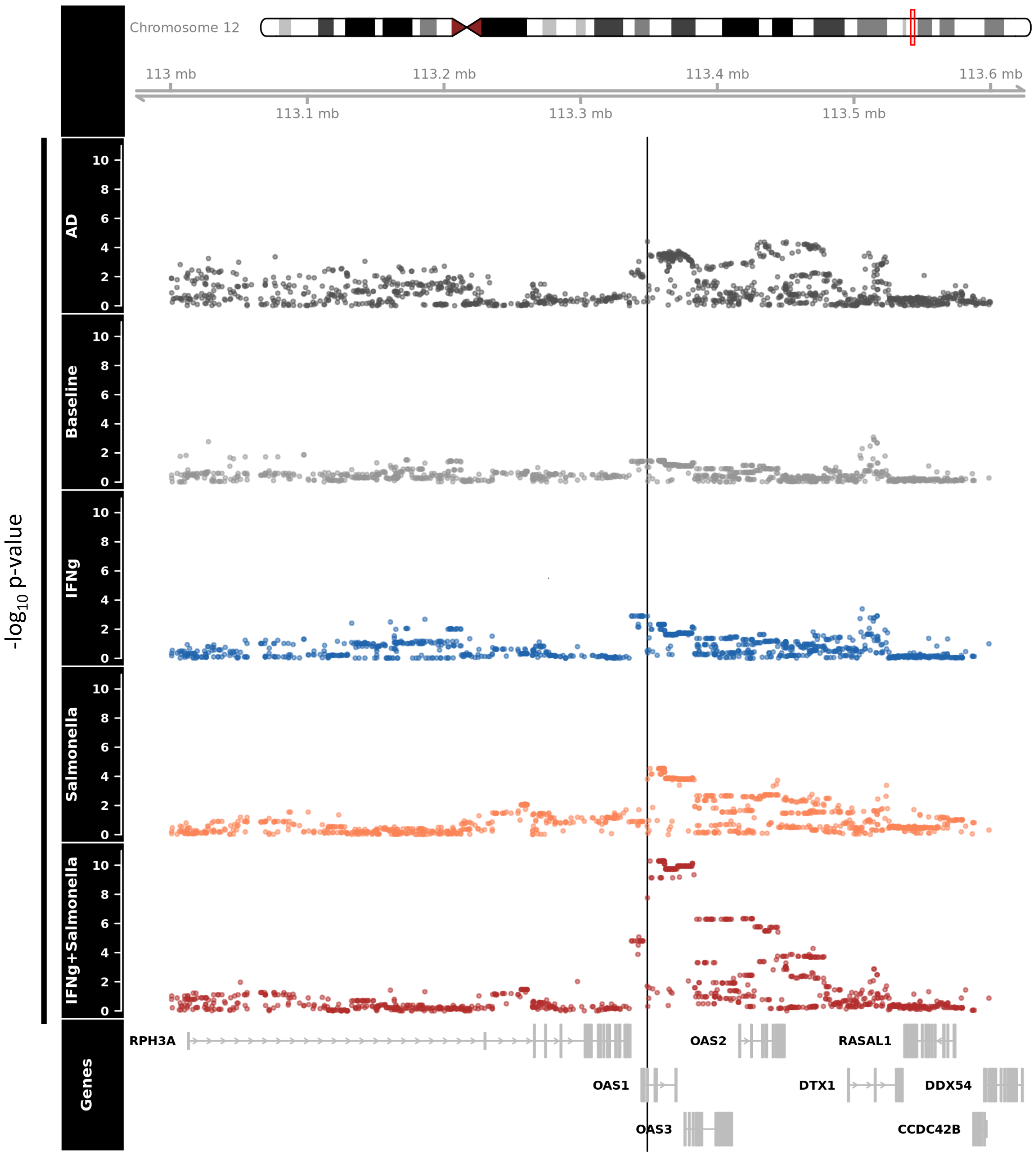

Supplementary Figure 8

Supplementary Figure 8 Colocalization of AD GWAS loci with eQTLs derived from baseline and stimulated iPSC-derived macrophages

Colocalization of AD loci and eQTLs targeting *OAS1* in baseline and stimulated states (IFNγ, 18 hours; Salmonella, 5 hours; IFNγ and Salmonella, 18 hours and 5 hours respectively). The eQTL data is from Alasoo *et al.*, (2018). The best Alzheimer's disease locus in *OAS1* from the IGAP data (Lambert *et al.*, 2013) is highlighted with the black line. IFNγ, interferon-γ. Numerical results are reported in Supplementary Table 4.

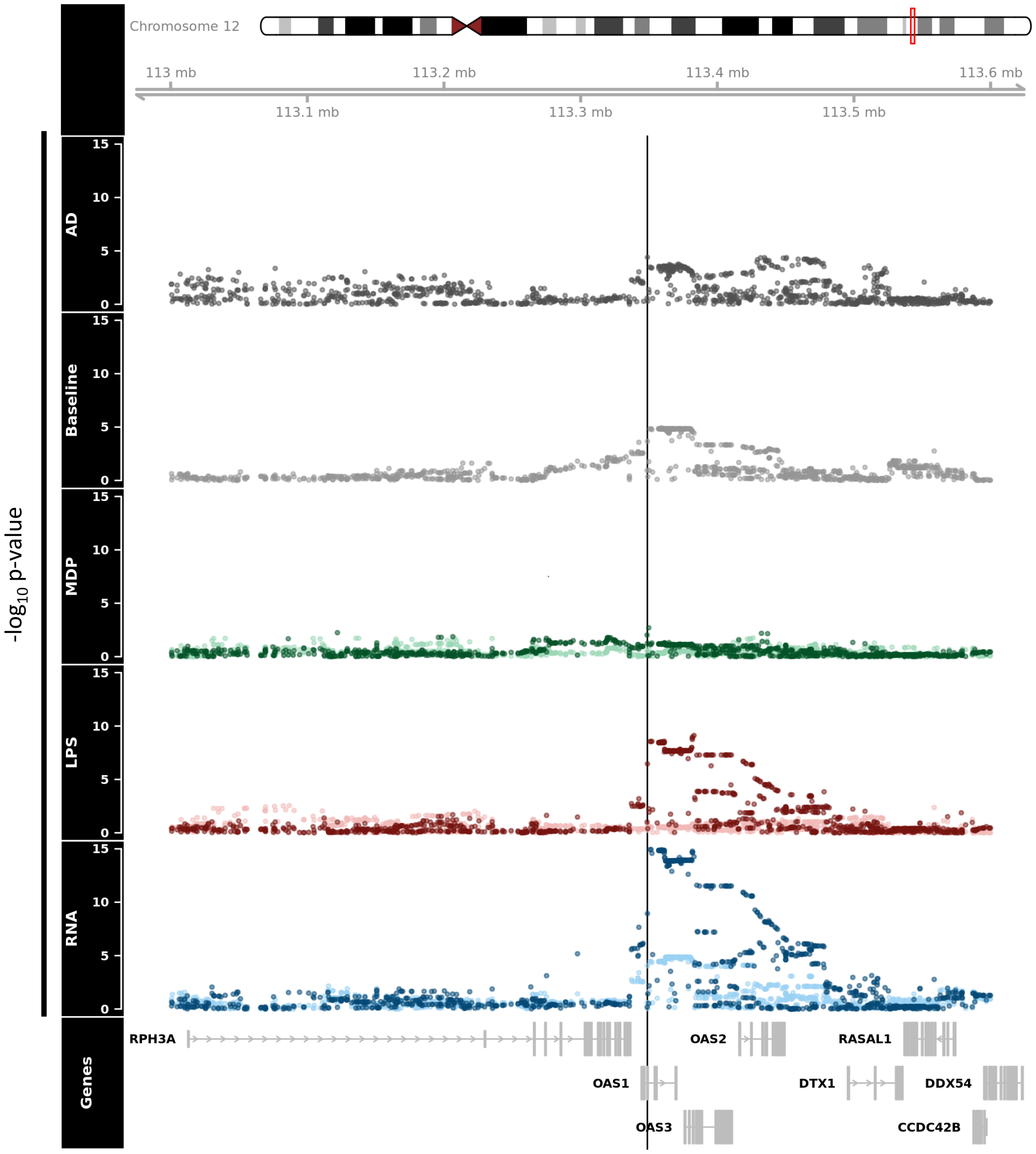

Supplementary Figure 9

Supplementary Figure 9 Colocalization of AD GWAS loci with eQTLs derived from baseline and stimulated human-derived monocytes

Colocalization of AD loci and eQTLs targeting *OAS1* in baseline and stimulated states. In MDP, LPS and RNA panels, lighter and darker data points represent monocytes stimulated for 90 minutes and 6 hours, respectively. The eQTL data is from Kim-Hellmuth *et al.*, (2017). The best Alzheimer's disease locus in *OAS1* from the IGAP data (Lambert *et al.*, 2013) is highlighted with the black line. LPS, lipopolysaccharide; MDP, muramyl-dipeptide; RNA, 5-triphosphate RNA. Numerical results are reported in Supplementary Table 4.

Supplementary Table 1 All genes in innate immune module associated with amyloid deposition

Available to download.

Supplementary Table 2 The mouse genes showing the tightest expression in amyloid-responsive microglia, which form the innate immune module plotted in Figure 1 (TOM > 0·39)

Available to download.

Supplementary Table 3 Putative risk genes in entire immune module associated with amyloid pathology (entire immune module in Supplementary Table 1)

Available to download.

Supplementary Table 4 Colocalization of Alzheimer's disease GWAS loci with eQTLs derived from baseline and stimulated iPSC-derived macrophages and human-derived monocytes

Available to download.

Supplementary Table 5 Human microglial genes expressed preferentially in purified human microglia (Galatro *et al.,* 2017 and Gosselin *et al.,* 2017), but orthologues not present in the mouse co-expression network present in amyloid-responsive microglia (Supplementary Table 1)

Available to download.
